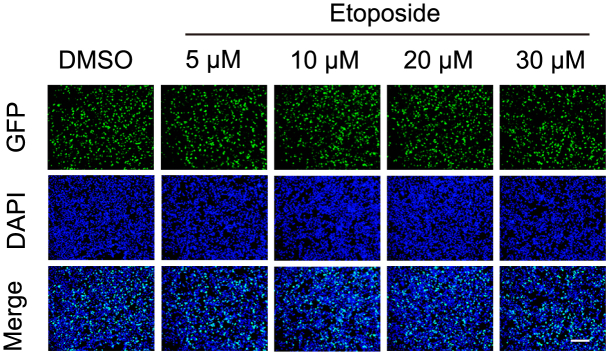# Identification of a conserved G-quadruplex within the E165R of African swine fever virus (ASFV) as a potential antiviral target

**DOI:** 10.1016/j.jbc.2024.107453

**Published:** 2024-06-07

**Authors:** Wenhao Liu, Xinglin He, Yance Zhu, Yaqin Li, Zhihao Wang, Pengfei Li, Jiajia Pan, Jiang Wang, Beibei Chu, Guoyu Yang, Mengjia Zhang, Qigai He, Yongtao Li, Wentao Li, Chao Zhang

**Affiliations:** 1 College of Veterinary Medicine, Henan Agricultural University, Zhengzhou, China; 2 Key Laboratory of Animal Biochemistry and Nutrition, Ministry of Agriculture and Rural Affairs, Zhengzhou, China; 3 Key Laboratory of Animal Growth and Development of Henan Province, Henan Agricultural University, Zhengzhou, China; 4 National Key Laboratory of Agricultural Microbiology, College of Veterinary Medicine, Huazhong Agricultural University, Wuhan, China; 5 Hubei Hongshan Laboratory, Wuhan, China

**Keywords:** African swine fever virus, ASFV, G-quadruplex, G4 ligands, antiviral activity

## Abstract

Identification of a conserved G-quadruplex in E165R of ASFVAfrican swine fever virus (ASFV) is a double-stranded DNA arbovirus with high transmissibility and mortality rates. It has caused immense economic losses to the global pig industry. Currently, no effective vaccines or medications are to combat ASFV infection. G-quadruplex (G4) structures have attracted increasing interest because of their regulatory role in vital biological processes. In this study, we identified a conserved G-rich sequence within the E165R gene of ASFV. Subsequently, using various methods, we verified that this sequence could fold into a parallel G4. In addition, the G4-stabilizers pyridostatin and 5,10,15,20-tetrakis-(N-methyl-4-pyridyl) porphin (TMPyP4) can bind and stabilize this G4 structure, thereby inhibiting E165R gene expression, and the inhibitory effect is associated with G4 formation. Moreover, the G4 ligand pyridostatin substantially impeded ASFV proliferation in Vero cells by reducing gene copy number and viral protein expression. These compelling findings suggest that G4 structures may represent a promising and novel antiviral target against ASFV.

African swine fever, caused by African swine fever virus (ASFV), is an infectious disease of swine that was first identified in Kenya in 1921 (1) and rapidly spread to most countries of sub-Saharan Africa (2). ASFV can be transmitted through contact with contaminated pork products and tick bites, resulting in rapid and severe damage, such as decreased appetite, difficulty breathing, and persistent fever. The mortality rate caused by ASFV infection can reach 100% (3, 4). Currently, ASFV is endemic to Europe, Asia, and Africa (5), causing substantial economic losses to the global pig industry. However, there is currently no commercial vaccine or medication available to control and prevent ASFV spread (6, 7). The genome of ASFV is composed of double-stranded DNA with a length of 170 to 190 kb, which encodes more than 60 structural proteins and over 100 nonstructural proteins (8, 9, 10). Notably, among the nonstructural proteins, E165R, K196R, and A240L play crucial roles as nucleic acid metabolic enzymes, providing energy during the replication process of the virus (11, 12).

The E165R protein functions as deoxyuridine 5′ triphosphate nucleotidohydrolase(dUTPase), which plays a vital role in maintaining the fidelity of DNA replication through two distinct mechanisms. Firstly, dUTPase hydrolyzes cellular dUTP into dUMP and pyrophosphate, thereby preventing the misincorporation of dUTP into synthesized DNA by DNA polymerase, which could lead to mutagenesis (13). Secondly, dUTPase provides the essential substrate, dUMP, to thymidylate synthase for the synthesis of dTTP (8, 14). The significance of dUTPase has been demonstrated in various viruses, including the vaccinia virus (11), herpes simplex virus type 1(HSV-1) (12), and white spot syndrome virus (15). Additionally, several studies have explored the structure and catalytic mechanism of E165R, which have provided valuable insights for drug development targeting dUTPase (8, 13, 14, 16). Given that E165R plays a crucial role in maintaining the fidelity of the viral genome by reducing the dUTP/dTTP ratio, and a previous study showed that deletion of ASFV dUTPase can impede efficient ASFV replication in macrophages (17), suggesting that E165R may serve as a potential antiviral target against ASFV.

G-quadruplexes (G4s) are noncanonical secondary structures formed by G-rich DNA or RNA. The G4 structure consists of stacking tetrads composed of four guanine bases connected by Hoogsteen hydrogen bonds (18, 19). Growing evidence indicates the presence of G4s in viral genomes, including pseudorabies virus (20, 21, 22, 23), HIV (24), Epstein-Barr virus (25), human papillomavirus (26), hepatitis B virus (27), HSV (28), Nipah virus (29), hepatitis C virus (HCV) (30), Zika virus (31, 32), Ebola virus (33), chikungunya virus (34), enterovirus A71 (35), Kaposi’s sarcoma-associated herpes virus (36), varicella-Zoster virus (37), severe acute respiratory syndrome (SARS)-CoV (38), and SARS-CoV-2 (39, 40). These G4 structures play vital roles in regulating virus replication, transcription, and translation. Moreover, several G4 stabilizers have been developed that preferentially bind to G4 structures, enhancing their thermal stability. These stabilizers have the potential to affect the viral life cycle by binding and stabilizing the G4 structures of the viruses, thus making them possible candidates for new antiviral agent development (41).

In this study, through bioinformatic analysis, we identified a G4 structure consisting of three G-tetrads within the coding region of E165R in ASFV. Biophysical and biochemical techniques collectively demonstrated that this G4 structure could adopt a parallel conformation. The G4 stabilizers, such as pyridostatin (PDS) and 5,10,15,20-tetrakis-(N-methyl-4-pyridyl) porphin (TMPyP4), significantly enhanced the thermal stability of the G4 structure and impeded the movement of Taq polymerase along templates containing the G4 region. Furthermore, an enhanced green fluorescent protein (EGFP) reporter assay indicated that this G4 structure could impede gene expression. Moreover, the G4 stabilizers PDS and TMPyP4 were found to inhibit E165R expression at the posttranscriptional level. Importantly, PDS significantly inhibited ASFV replication in Vero cells by reducing viral genome copy numbers and protein synthesis. These findings suggest that the G4 structure present in E165R could serve as a novel target for anti-ASFV strategies.

## Results

### A G-rich sequence within the coding sequence region of the E165R gene forms a parallel G4 structure *in vitro*

**Figure 1:**
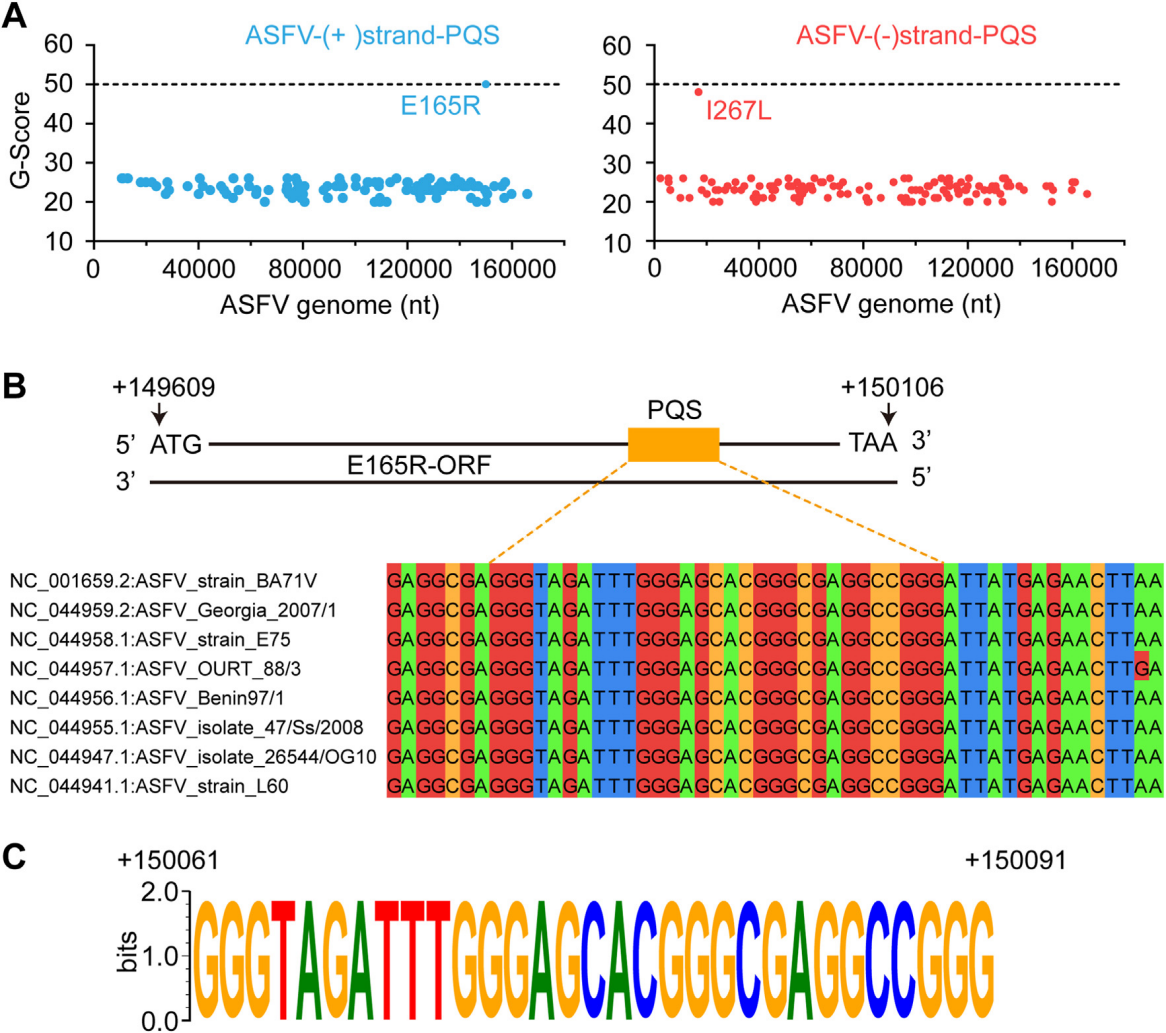
**Identification of the G4-forming sequence in the ASFV genome.***A*, both strands of the ASFV genome were subjected to bioinformatic analysis by using QGRS. Each *blue* and *red dot* corresponds to a G4-forming sequence located in the sense strand (+) and anti-sense strand (−), respectively. The G-score was plotted against the genome length. *B*, the schematic map of E165R's G4 distribution, and the genome of eight representative ASFV strains were aligned with Jalview. *C*, the conserved G4 motif was created by WebLogo software. ASFV, African swine fever virus; G4, G-quadruplex.

A previous study reported that G4s with short loops exhibit better stability than those with long loops (42, 43). To identify potential quadruplex-forming sequences (PQSs) within the ASFV genome, we utilized the online software QGRS Mapper (https://bioinformatics.ramapo.edu/QGRS/index.php) with stringent criteria: the guanine repeat number was set at 2, and the loop length was varied from 1 to 7 nt (44). As a result, we identified 119 PQSs that were distributed in the sense strand (+) of the ASFV genome (listed in Table S1), while 149 PQSs located in the anti-sense strand (−) were identified (listed in Table S2). Most of these PQSs comprised of two-layer quartets, with G-scores averaging around 25. Notably, we identified a prominent PQS with the highest G-score of 50 in the coding region of E165R (+149609 to +150106), while a G-score of 48 was identified in the I267L gene (Fig. 1, *A* and *B*). In addition, we used another widely used algorithm, pqsfinder, to cross-check the PQS on both strands of ASFV. Similar to the results obtained from QGRS Mapper, two PQS with high scores were identified in E165R and I267L (Table S3). The G-scores derived from pqsfinder and the QGRS Mapper were different, which could be ascribed to the different scoring rules used by the two algorithms. Moreover, multiple sequence alignment analysis revealed that the PQS of E165R was conserved among 55 different strains (Fig. 1*C*).

**Figure 2:**
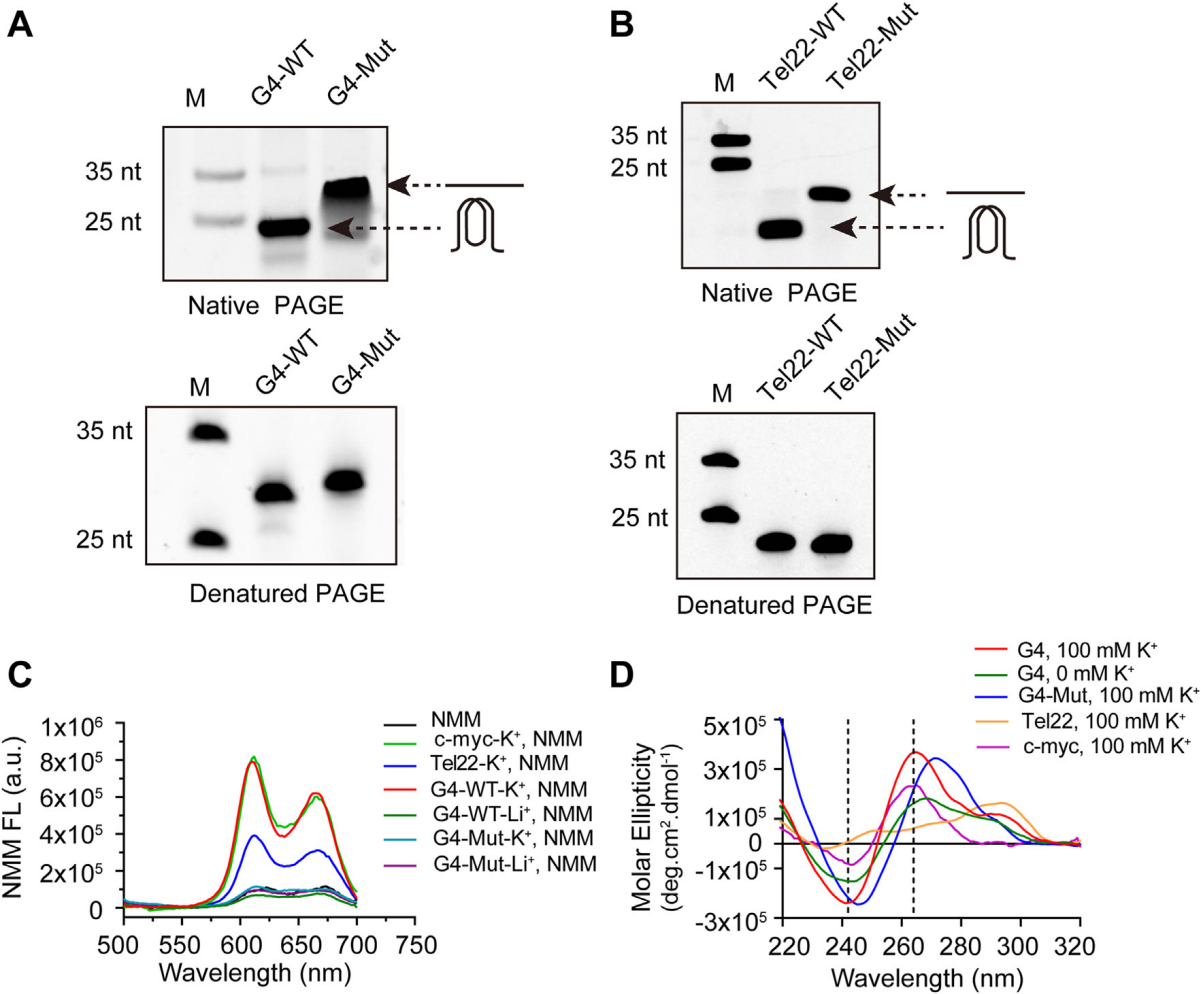
**Characterization of G4 formation of E165R G4 DNA.***A*, oligonucleotides of G4-WT DNA and G4-Mut were heated at 9 °C for 3 min and then slowly cooled to 25 °C. The sample was subjected to native PAGE analysis in which the buffer contained 100 mM K+, or to denatured PAGE containing 7 M urea. Then, the gel was imaged with a GE AI600 imaging system. *B*, human telomere sequence (Tel22) was used as a positive control for E165R G4 DNA. *C*, G4-WT, c-myc, Tel22, and G4-Mut were annealed in the presence of K+ or the presence of Li+, and then NMM was added. The fluorescence of NMM was recorded using a SpectraMax i3X multifunctional microplate reader, the excitation wavelength was set to 393 nm, and the emission spectrum was collected in the range of 450 nm to 700 nm. *D*, G4-DNA was annealed in the presence and absence of 100 mM k+, followed by CD scanning. G4-Mut was used as a negative control, while c-myc and Tel22 served as positive control. G4, G-quadruplex; NMM, N-methyl mesoporphyrin IX.

To assess G4 formation, we initially used native PAGE since previous studies have demonstrated that oligonucleotides containing intramolecular G4 structures migrate faster than their linear counterparts in native PAGE. 6-carboxyfluorescein (FAM) was labeled at the 5′ end of the oligonucleotide for visualization purposes. As depicted in Figure 2*A*, G4 DNA migrated faster than the mutated control, in which specific G residues were mutated to A to prevent G4 formation. This observation indicates that G4 DNA folds into an intramolecular G4 structure in the presence of 100 mM K+. Moreover, the differential migration rates between G4 DNA and G4-mutated DNA cannot be attributed to mass weight differences, as both G4 DNA and G4-mutated DNA exhibited similar migration in denaturing PAGE with urea. This result was similar to that of a well-studied G4-forming sequence Tel22(Fig. 2*B*).

To further confirm G4 formation in E165R-G4-DNA, we performed a fluorescent turn-on assay using N-methyl mesoporphyrin IX (NMM) (45), a fluorescent compound that binds to G4 structures. Compared to NMM alone, the presence of G4 DNA in 100 mM K+ (which stabilizes the G4 structure) resulted in enhanced fluorescence, which was similar to that of the positive controls Tel22 and c-myc. In comparison, at 100 mM Li+ (which does not stabilize G4), the NMM did not exhibit increased fluorescence even in the presence of G4 DNA. As a negative control, G4-Mut failed to enhance NMM fluorescence under K+ and Li+ conditions (Fig. 2*C*).

Finally, CD experiments were conducted to validate G4 formation collectively. The CD is a standard method for analyzing G4 folding and topology (46). C-myc and Tel22 were used as positive controls to revalidate our CD assay. Upon annealing in 100 mM K+, G4 DNA displayed a negative peak near 240 nm and a positive peak around 263 nm, characteristic of a parallel G4 conformation, whereas G4-Mut did not exhibit these features. Furthermore, reducing the K+ concentration decreased the peak intensity associated with G4 topology (Fig. 2*D*).

In summary, these results collectively demonstrate that the G4 DNA within the coding region of E165R can fold into an intramolecular G4 structure.

### The G4 stabilizer was able to bind and stabilize the G4 structure of E165R-G4

**Figure 3:**
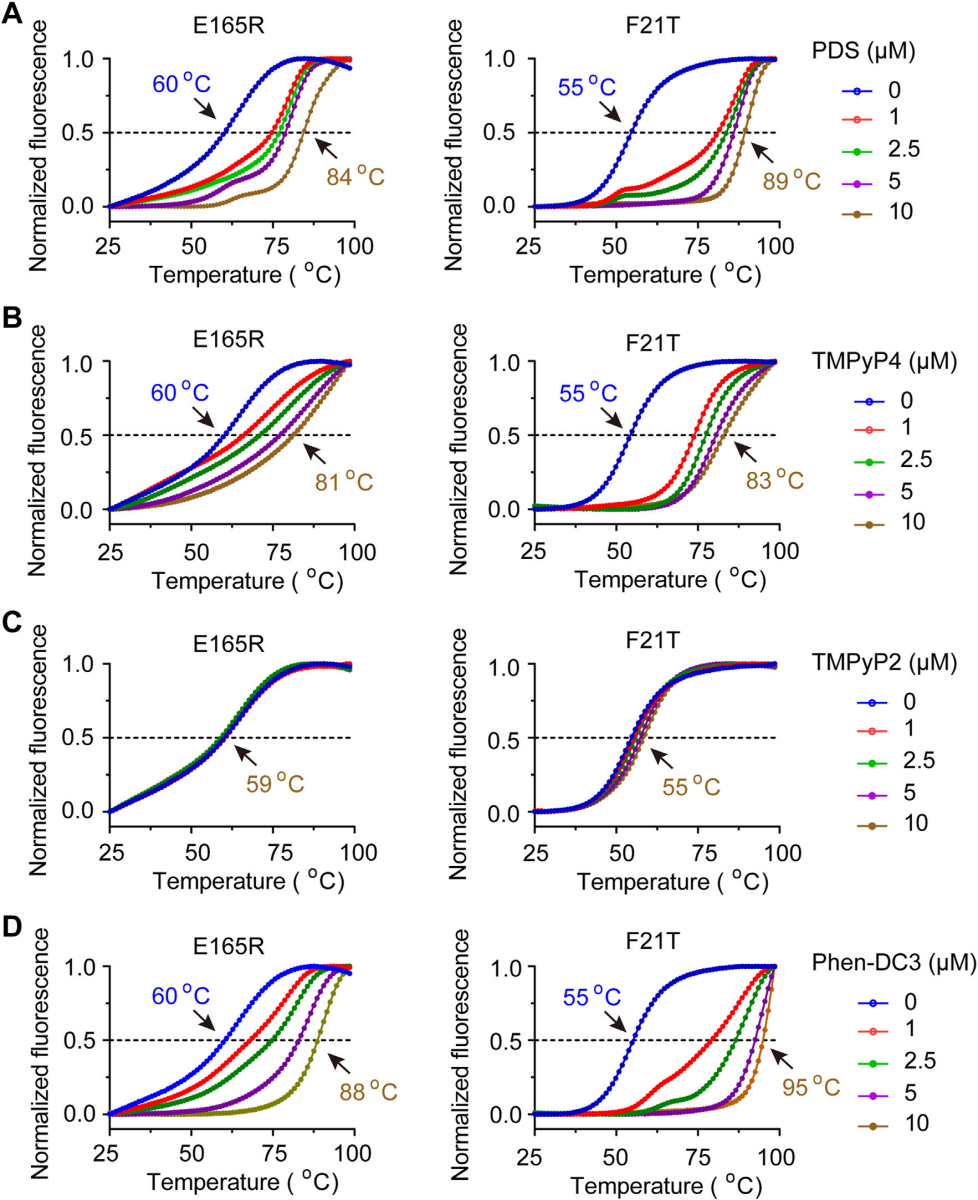
**G4 ligands stabilize the G4 structure formed in G4-DNA of E165R.***A*–*D*, the G4-DNA of E165R or F21T was annealed in the presence of 100 mM k+, followed by the addition of different concentrations of ligand (0 μM, 1 μM, 2.5 μM, 5 μM, and 10 μM) at 25 °C for 30 min. The 6-carboxyfluorescein fluorescence was recorded, and the normalized 6-carboxyfluorescein fluorescence was plotted against the various temperatures. The *dotted line* indicates the middle of the maximum fluorescence and represents the Tm of the G4 structure. G4, G-quadruplex.

The ligand PDS, TMPyP4, Phen-DC3 (3,3′-[1,10-Phenanthroline-2,9-diylbis(carbonylimino)]bis [1-methylquinolinium]1,1,1-trifluoromethanesulfonate(1:2)), are well-known small molecule compounds that specifically target and stabilize G4 structures (47, 48, 49). On the other hand, TMPyP2, an isomer of TMPyP4, exhibits a low affinity for G4 structures (50). To investigate the binding interaction between PDS and TMPyP4 with E165R-G4, we conducted a FRET melting experiment. Specifically, we labeled FAM and tetramethylrhodamine (TAMRA) at the 5′ and 3′ ends of the E165R-G4 oligonucleotide, respectively. The proximity of TAMRA to FAM leads to the suppression of FAM fluorescence when the oligonucleotide adopts a G4 structure. As the temperature increases, the G4 structure unfolds, resulting in an increase in the distance between FAM and TAMRA, thus allowing the fluorescence of FAM to be detected. E165R-G4 was annealed in 100 mM K+ to promote G4 folding and then incubated with PDS, TMPyP4, TMPyP2, or Phen-DC3 at 25 °C for 30 min. The fluorescence emitted by FAM was recorded, and the temperature at which the fluorescence intensity reached half of its maximum value was defined as the melting temperature. As shown in Figure 3*A*, the melting temperature of E165R-G4 increased from 60 °C to 84 °C in the presence of PDS, compared to the negative control without PDS. Similarly, the melting temperature of E165R-G4 increased from 60 °C to 81 °C for TMPyP4, and from 60 to 88 °C for Phen-DC3 (Fig. 3, *B* and *D*) In contrast, the compound TMPyP2 which served as a negative control for TMPyP4, did not alter the melting temperature of E165R-G4 (Fig. 3*C*). Meanwhile, the FRET melting experiment with a telomere sequence (F21T) confirmed the reliability of our experiment. These results indicate that PDS, TMPyP4, and Phen-DC3 can effectively bind to and stabilize the G4 structure formed by E165R-G4.

### G4 ligands inhibit DNA synthesis at G4 sites

**Figure 4:**
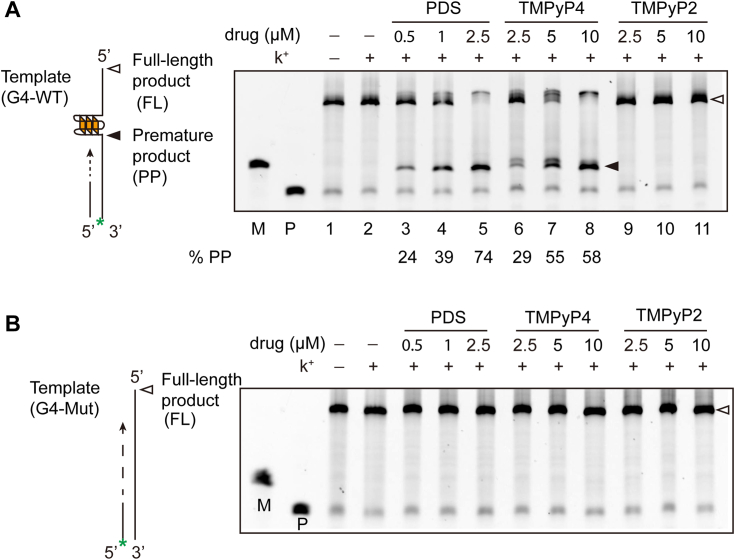
**G4 ligands inhibit DNA synthesis at the G4 sites.***A*, schematic illustration of the Taq polymerase termination assay, where the 5′-6-carboxyfluorescein-labeled primer was complementary to the template DNA harboring the G4 motif and then extended by Taq polymerase in the presence of different ligands. The extension product was run in denatured PAGE with 7M urea, and the gel was imaged with an Amersham Imager 600. The *black arrow* indicates the premature product (pp) caused by G4, while the *white arrow* indicates the full-length product (FL). % pp was calculated by intensity (PP)/[intensity (PP)+intensity (FL)]. *B*, the template with the G4-Mut counterpart was subjected to a Taq polymerase assay as shown in Figure 4*A*. G4, G-quadruplex.

To further investigate the biological function of the G4 structure, we performed a Taq polymerase termination assay to mimic the DNA replication process. In this assay, a single-stranded DNA template containing either the G4 or G4 mut sequence was amplified using Taq polymerase in the presence or absence of various G4 stabilizers. Only the full extension product was observed when the reaction was conducted in the absence of K+ (Fig. 4*A*, lane 1). However, upon the addition of K+ and PDS, which stabilize the G4 structure, a distinct stop band below the full-length band was observed at specific G4 sites, except for the full-length product (Fig. 4*A*, lane 3), and more termination products were observed when increasing amounts of PDS added. Specifically, treatment with 2.5 μM PDS increased the stop product to approximately 74%.

Similar to PDS treatment, TMPyP4 treatment also induced Taq polymerase termination at the G4 sites, yielding more termination products (Fig. 4*A*, lanes 6, 7, and 8). As a negative control for TMPyP4, TMPyP2 did not inhibit Taq polymerase extension. In contrast, the single-stranded DNA template containing the G4 mut motif failed to produce premature termination products, even in the presence of K+ and ligands (Fig. 4*B*).

These findings indicate that the G4 stabilizers PDS and TMPyP4 effectively inhibit Taq polymerase extension by stabilizing the G4 structure.

### G4 RNA of the E165R gene can form RNA G4 structure

**Figure 5:**
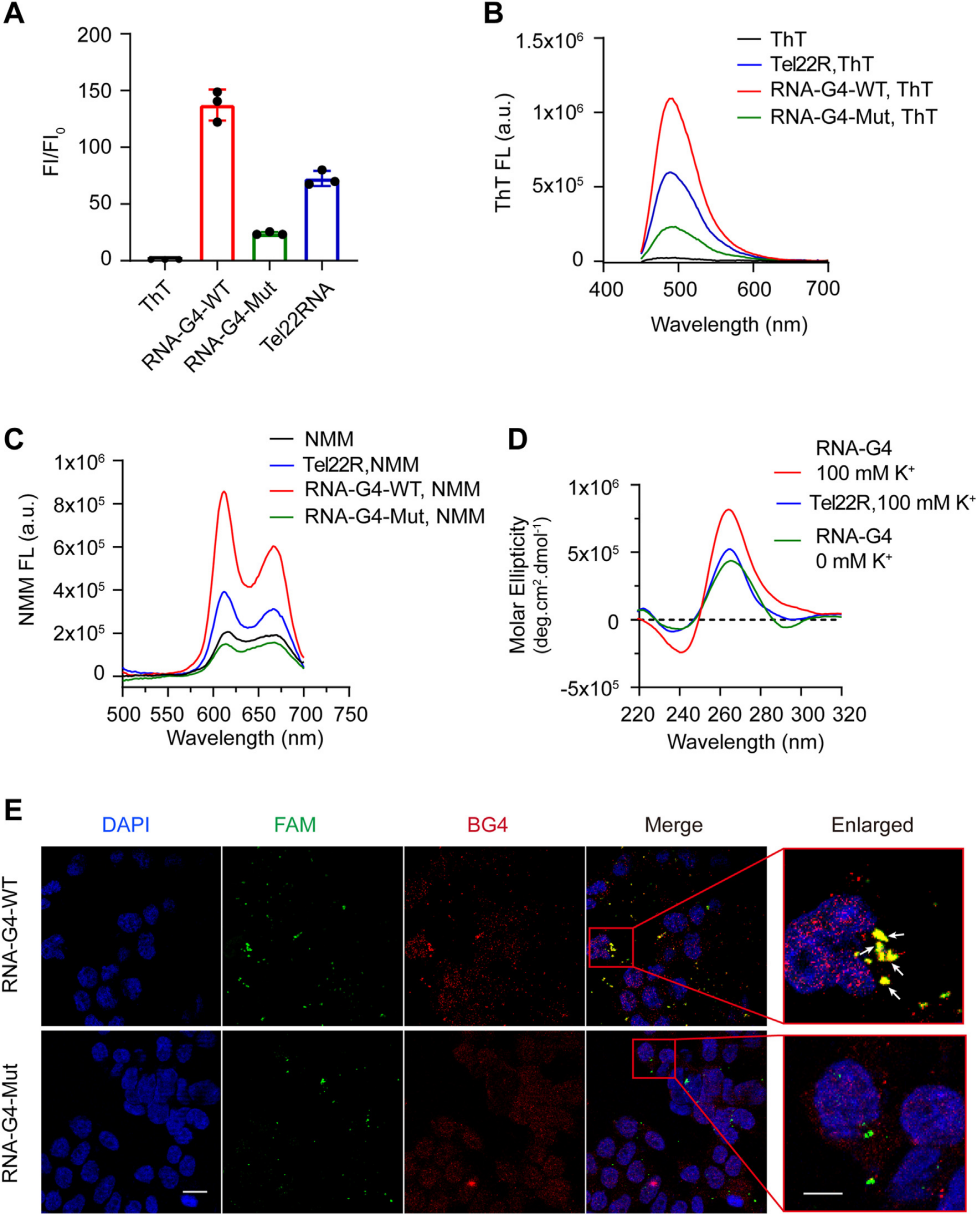
**Characterization of RNA G4 formation.***A*, the RNA oligonucleotide corresponding to the G4 DNA of E165R was annealed in 100 mM K+ and then incubated with ThT. The excitation and emission wavelengths were 425 nm and 495 nm, respectively. The changed fluorescence of ThT plotted to different oligonucleotides was calculated by FI/FI_0_, where FI and FI_0_ indicated sample and background fluorescence, respectively. *B*, the fluorescence spectrum of ThT as shown in Figure 5*A*. *C*, fluorescence spectrum of NMM in the presence of RNA-G4-WT or RNA-G4-Mut annealed in 100 mM K+. *D*, CD spectral analysis for rG4 in the presence and absence of 100 mM k+. The Tel22R was used as a positive control in Figure 5, *A*–*D*. *E*, FAM-labeled RNA-G4-WT and G4-Mut were transfected into 293T cells, BG4 antibody (*red*) was applied to probe G4 formation, and the nuclei were stained with DAPI. Colocalization of G4-WT and BG4 is indicated by the *white arrow*. The scale bar on the original image was 10 μm, and the scale bar on the enlarged image was 20 μm. DAPI, 4′,6-diamidino-2-phenylindole; G4, G-quadruplex; NMM, N-methyl mesoporphyrin IX.

Previous studies have reported that G-rich sequences within the coding region can form RNA G4 (rG4) structures when transcribed (51, 52, 53). Therefore, we were encouraged to examine whether the RNA version of G4 DNA in E165R can fold into rG4 structure, and the well-studied PQS Tel22 was used as positive control. The fluorescence turn-on assay was initially used for this purpose. As shown in Figure 5*A*, titration of RNA-G4-WT with ThT resulted in a significant 6-fold increase in fluorescence compared to the negative control, RNA-G4-Mut. This observation was further supported by the scanning spectrum of ThT when incubated with RNA-G4-WT (Fig. 5*B*). The NMM turn-on assay also yielded similar results to the ThT assay, wherein adding RNA-G4-WT led to enhanced fluorescence compared to the negative control, RNA-G4-Mut (Fig. 5*C*). Additionally, the CD spectrum results indicated that the rG4 could adopt a parallel G4 structure, as evidenced by the characteristic spectrum showing a positive peak at 260 nm and a negative peak at 240 nm. Furthermore, the intensity of the CD signal decreased when the concentration of K+ ions was reduced to 0 mM (Fig. 5*D*).

Based on the results above, which demonstrated that G4 RNA can fold into G4 structures under buffer conditions, we investigated the rG4 structure formation in cellular environments. To this end, FAM-labeled RNA-G4-WT and RNA-G4-Mut were transfected into 293T cells, and the G4-specific antibody BG4 was used to probe the presence of G4 structures. As presented in Figure 5*E*, the colocalization of discrete and punctate red signals with green signals resulted in yellow fluorescence, indicating the formation of G4 structures by RNA-G4-WT. In contrast, no overlapping signals were detected for RNA-G4-Mut.

Collectively, these findings provide compelling evidence that G4 RNA derived from E165R can fold into rG4 structures.

### G4 stabilizers suppress EGFP reporter gene expression

**Figure 6:**
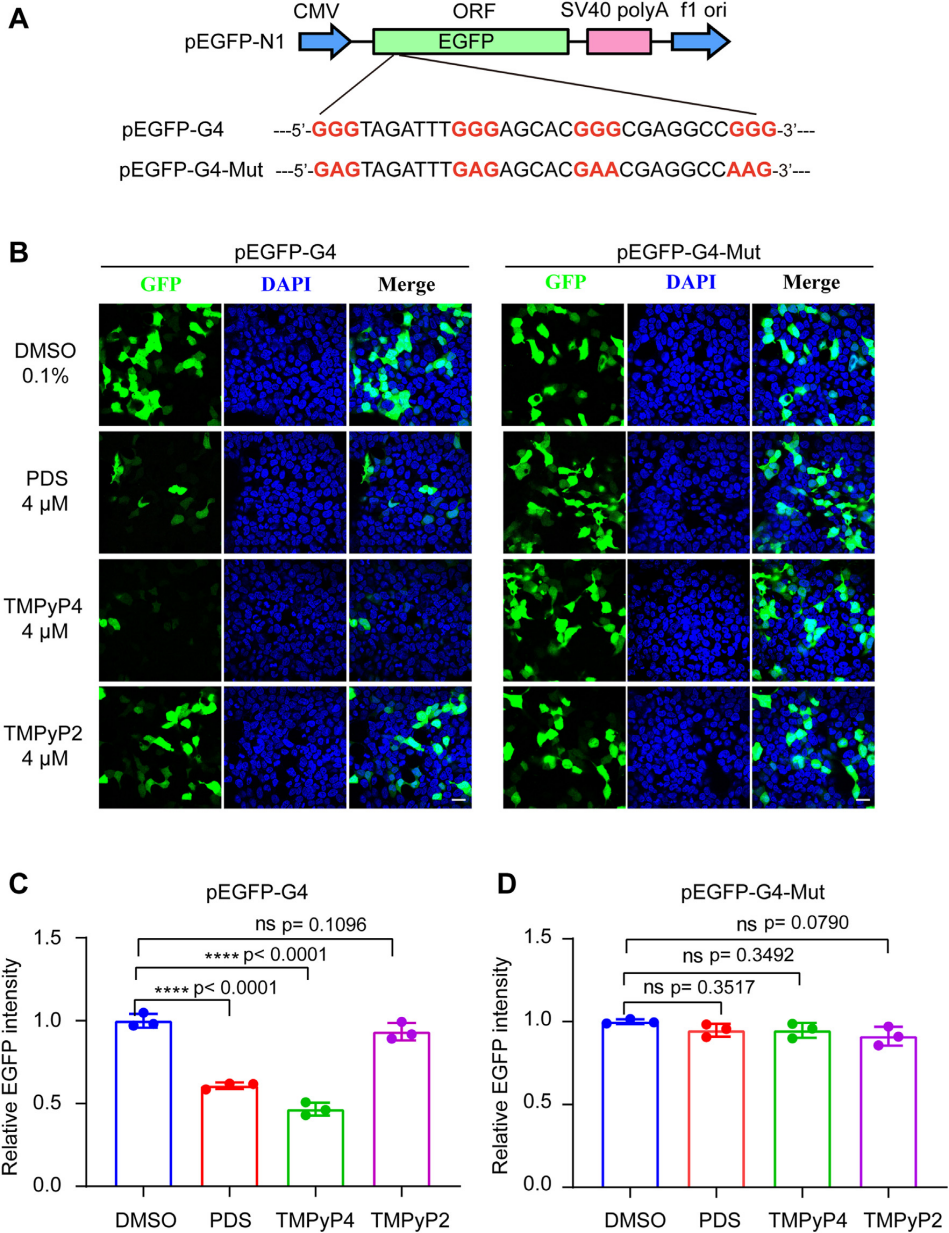
**G4 stabilizers repress EGFP reporter expression.***A*, schematic illustration of the reporter plasmid, where G4-WT and its counterpart G4-Mut were cloned into the coding sequence of EGFP. *B*, cells were pretreated with different ligands and then transfected with the reporter constructs, and EGFP was detected using a confocal microscope. *C* and *D*, quantified analysis of EGFP with flow cytometry. DMSO-treated samples were used as the negative control. All experiments were performed in triplicate. Data were analyzed by One-way ANOVA with Dunnett's multiple comparisons test, ∗∗∗ *p* < 0.001. ns, not significant. The scale bar was 20 μm. DMSO, dimethyl sulfoxide; EGFP, enhanced green fluorescent protein; G4, G-quadruplex.

**Figure S1:**
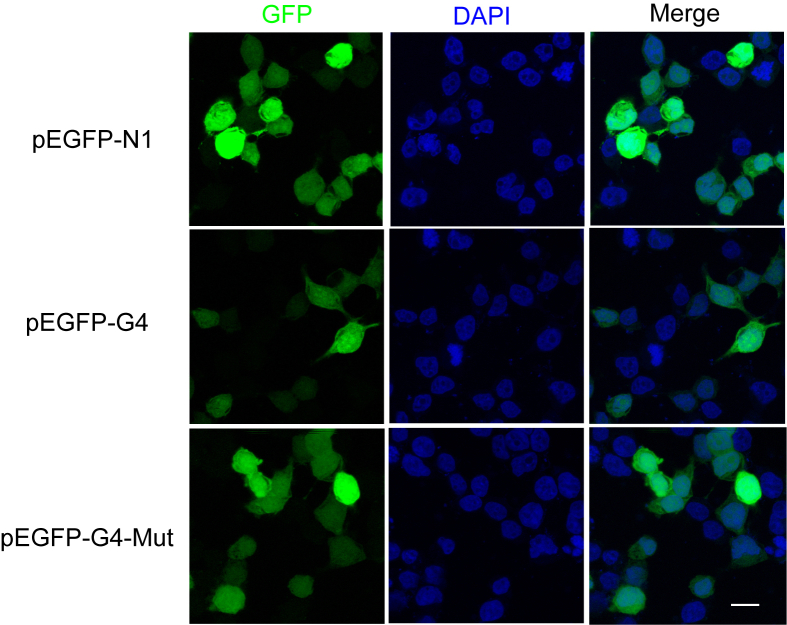


To explore the functional significance of the G4 structure formed in the E165R gene, an EGFP reporter gene system was used. Specifically, the E165R-G4 or E165R-G4-Mut sequence was inserted downstream of the EGFP start codon in the pEGFP-N1 vector, generating pEGFP-G4 and pEGFP-G4-Mut plasmids, respectively (Fig. 6*A*), and the G4 sequence that was inserted downstream of the initiation codon of EGFP only translated to 11 amino acids, which would not disturb EGFP translation. These plasmids were then transfected into 293T cells, and the expression of EGFP was assessed through confocal microscopy, the pEGFP-G4 exhibited weaker fluorescence than that of pEGFP-G4-Mut due to the inhibitory effect of G4 on EGFP translation (Fig. S1). Notably, adding PDS substantially diminished EGFP expression compared to the sample without the ligand. Similarly, TMPyP4 also exhibited an inhibitory effect on EGFP expression. In contrast, TMPyP2, which served as a negative control for TMPyP4, did not impact EGFP expression. Notably, in the negative control plasmid where the G4 motif was mutated to disturb G4 formation, the fluorescence of EGFP was unimpaired regardless of the presence of PDS or TMPyP4 (Fig. 6*B*).

Furthermore, the expression of EGFP was quantitatively analyzed using flow cytometry. The results revealed that both PDS and TMPyP4 significantly inhibited the expression of EGFP in cells transfected with the pEGFP-G4 plasmid (Fig. 6*C*). In contrast, the expression of EGFP in the control plasmid remained largely unaffected (Fig. 6*D*).

These experiments provide compelling evidence that PDS and TMPyP4 exert their inhibitory effects on the expression of the EGFP reporter gene by targeting and stabilizing the G4 structure formed in the E165R gene.

### G4 stabilizers inhibit full-length E165R gene expression

**Figure 7:**
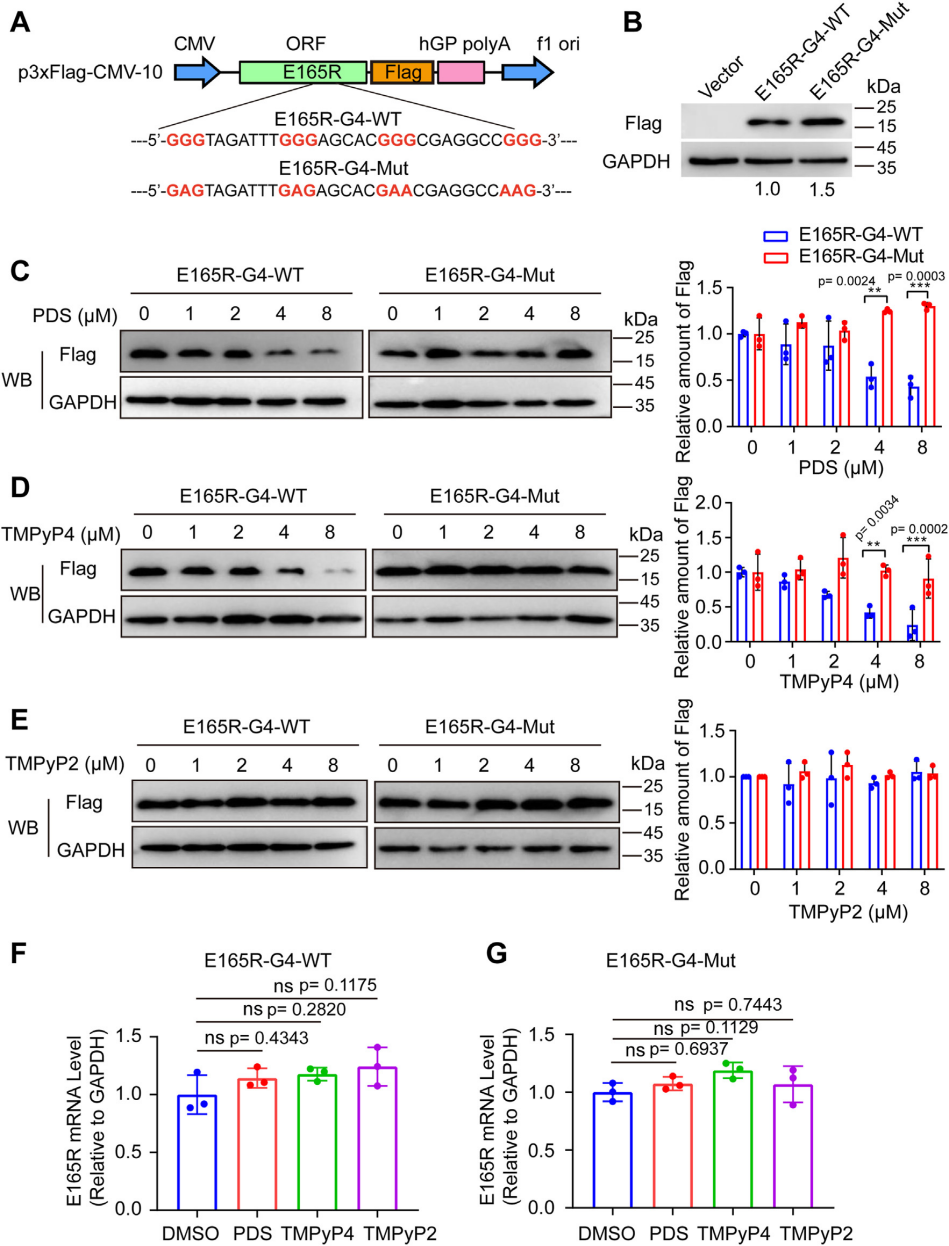
**G4 stabilizers suppress E165R expression.***A*, schematic representation of the plasmids used, in which the full-length E165R and the mutated counterpart where G was substituted with A to disturb G4 formation were inserted into the p3 × FLAG-CMV-10 vector. The verified plasmids were transfected into 293T cells and then cultured in medium supplemented with different concentrations of ligands for 24 h. The collected cells were lysed in radio immunoprecipitation assay lysis buffer and then subjected to Western blot analysis. *B*, the protein level of E165R-G4-WT and E165R-G4-Mut. *C*, the protein level of E165R-G4-WT and E165R-G4-Mutwhen treated with various concentrations of PDS (0 μM, 1 μM, 2 μM, 4 μM, and 8 μM). *D*, the protein level of E165R when treated with various concentrations of TMPyP4 (0 μM, 1 μM, 2 μM, 4 μM, and 8 μM). *E*, the protein level of E165R when treated with various concentrations of TMPyP2 (0 μM, 1 μM, 2 μM, 4 μM, and 8 μM). *F* and *G*, Q-PCR determined the RNA level of E165R-G4-WT and its mutated type after adding various ligands. All experiments were performed in triplicate. Data were analyzed by One-way ANOVA with Dunnett's multiple comparisons test, ∗∗*p* < 0.01, ∗∗∗∗ *p* < 0.0001. G4, G-quadruplex; ns, not significant; PDS, pyridostatin; TMPyP4, 5,10,15,20-tetrakis-(N-methyl-4-pyridyl) porphin.

Based on the aforementioned results, our next objective was to investigate whether G4 structures affect the expression of full-length E165R. To accomplish this, the complete coding sequence of E165R and its mutated counterpart where G4 was mutated were separately cloned into the p3 × FLAG-CMV-10 vector, resulting in the generation of the E165R-G4-WT and E165R-G4-Mut plasmids (Fig. 7*A*). Protein immunoblotting experiments were then performed to assess the impact of G4 structures and G4 stabilizers on E165R expression, E165R-G4-WT produced fewer proteins than the control samples where G4 was mutated (Fig. 7*B*).

The addition of PDS significantly inhibited E165R expression compared to the negative control without PDS. Notably, the suppressive effect of PDS on E165R expression was concentration-dependent (Fig. 7*C*). Similarly, TMPyP4 repressed the synthesis of E165R protein, similar to the effect of PDS (Fig. 7*D*). In contrast, TMPyP2 displayed no inhibitory effect (Fig. 7*E*). Importantly, in the case of the E165R-G4-Mut plasmid, where the G4 motif was mutated to disrupt G4 formation, no inhibitory effect was observed in the presence of either PDS or TMPyP4. These findings suggest that G4 structures play a role in stabilizing PDS and TMPyP4, thereby suppressing E165R expression at the protein level, and this effect is dependent on the presence of G4 structures.

As gene expression can be influenced at both the transcriptional and translational levels, we further explored which stage of E165R expression was affected by G4 DNA and G4 stabilizers. Interestingly, real-time quantitative reverse transcription PCR results revealed that neither PDS nor TMPyP4 impaired the synthesis of E165R mRNA when compared to the dimethyl sulfoxide (DMSO) control (Fig. 7*F*). Additionally, the E165R-G4-Mut sample exhibited similar results (Fig. 7*G*), indicating that G4 structures or G4 stabilizers did not impact E165R transcription.

These results suggest that G4 structures stabilize and hinder E165R expression at the posttranscriptional stage rather than affecting transcription.

### G4 stabilizers inhibit ASFV proliferation in cells

**Figure 8:**
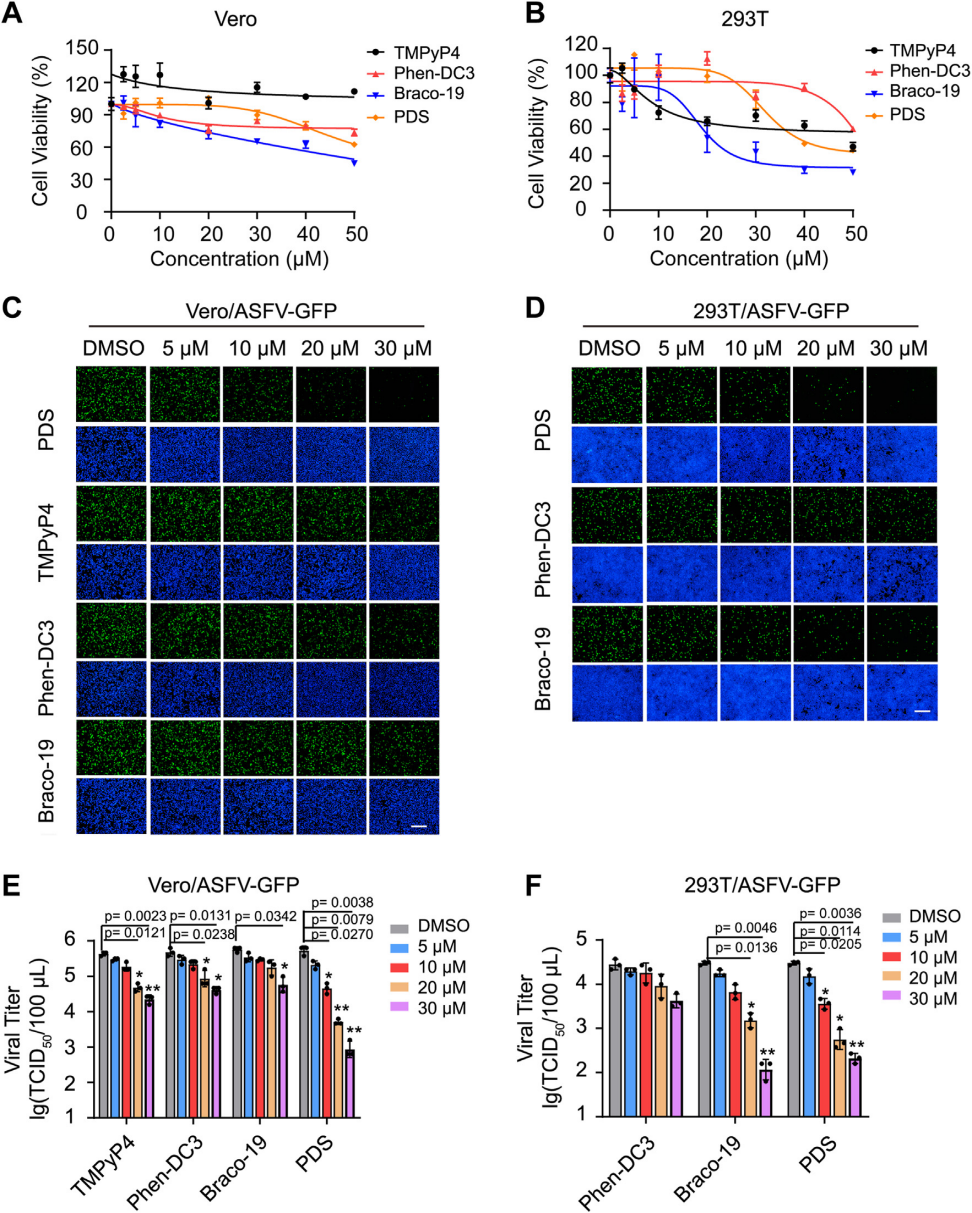
**Effects of G4 ligands on the proliferation of African swine fever virus in different cells.***A*, cytotoxicity of the four G4 ligands in Vero cells. *B*, cytotoxicity of the four G4 ligands in 293T cells. *C*, effects of the four G4 ligands on the proliferation of African swine fever virus in Vero cells. Vero cells were pretreated with the indicated G4 ligands and then infected with African swine fever virus (ASFV-GFP) at an MOI of 0.5, followed by the replacement of the medium containing the corresponding G4 ligands. After 36 h of infection, fluorescence was observed under a fluorescence microscope. *D*, effects of the four G4 ligands on ASFV proliferation in 293T cells. *E*, the inhibitory effect of the four G4 ligands on ASFV proliferation in Vero cells was determined by TCID_50_ assay. *F*, the inhibitory effect of the four G4 ligands in 293T cells was determined by TCID_50_ assay. All experiments were performed in triplicate. Data were analyzed by One-way ANOVA with Dunnett's multiple comparisons test, ∗*p* < 0.05, ∗∗*p* < 0.01. The scale bar was 200 μm. ASFV, African swine fever virus; G4, G-quadruplex; MOI, multiplicity of infection; TCID_50_, median tissue culture infectious dose.

Next, we aimed to investigate whether G4 stabilizers could impact ASFV proliferation in cells. In this study, four well-known G4 stabilizers, namely PDS, TMPyP4, Phen-DC3, and Braco-19, were used along with Vero and 293T cells, which have been used as cell models for ASFV infection (54, 55). Initially, the Cell Counting Kit-8 assay results demonstrated that, except for Braco-19, the other three compounds exhibited lower cytotoxicity to Vero cells when used at concentrations below 30 μM (Fig. 8*A*). Similarly, for 293T cells, Phen-DC3 and PDS displayed relatively lower cytotoxicity when used at concentrations below 30 μM, whereas TMPyP4 and Braco-19 showed greater cytotoxicity (Fig. 8*B*). Therefore, we selected a ligand concentration of 30 μM as the maximum concentration for subsequent experiments.

As shown in Figure 8*C*, PDS treatment led to a concentration-dependent decrease in ASFV-GFP-infected Vero cells, with significant suppression observed at 30 μM, as confirmed by immunofluorescence assay. Similar results were obtained for 293T cells, where PDS and Braco-19 substantially inhibited GFP fluorescence signal (Fig. 8*D*).

Furthermore, the median tissue culture infectious dose (TCID_50_) results for ASFV-infected Vero cells demonstrated that compared with DMSO treatment, PDS treatment reduced the viral titer by approximately two orders of magnitude. In contrast, TMPyP4, Phen-DC3, and Braco-19 had no significant impact on the viral titer (Fig. 8*E*). Similarly, in ASFV-infected 293T cells, Braco-19 and PDS exhibited inhibitory effects on ASFV proliferation (Fig. 8*F*). However, it should be noted that the inhibitory effect of Braco-19 was mainly due to its cytotoxicity on 293T cells.

Taken together, these findings suggest that G4 ligands, particularly PDS, can inhibit ASFV proliferation in Vero and 293T cells.

### PDS affects virus genome replication and protein synthesis of ASFV

**Figure 9:**
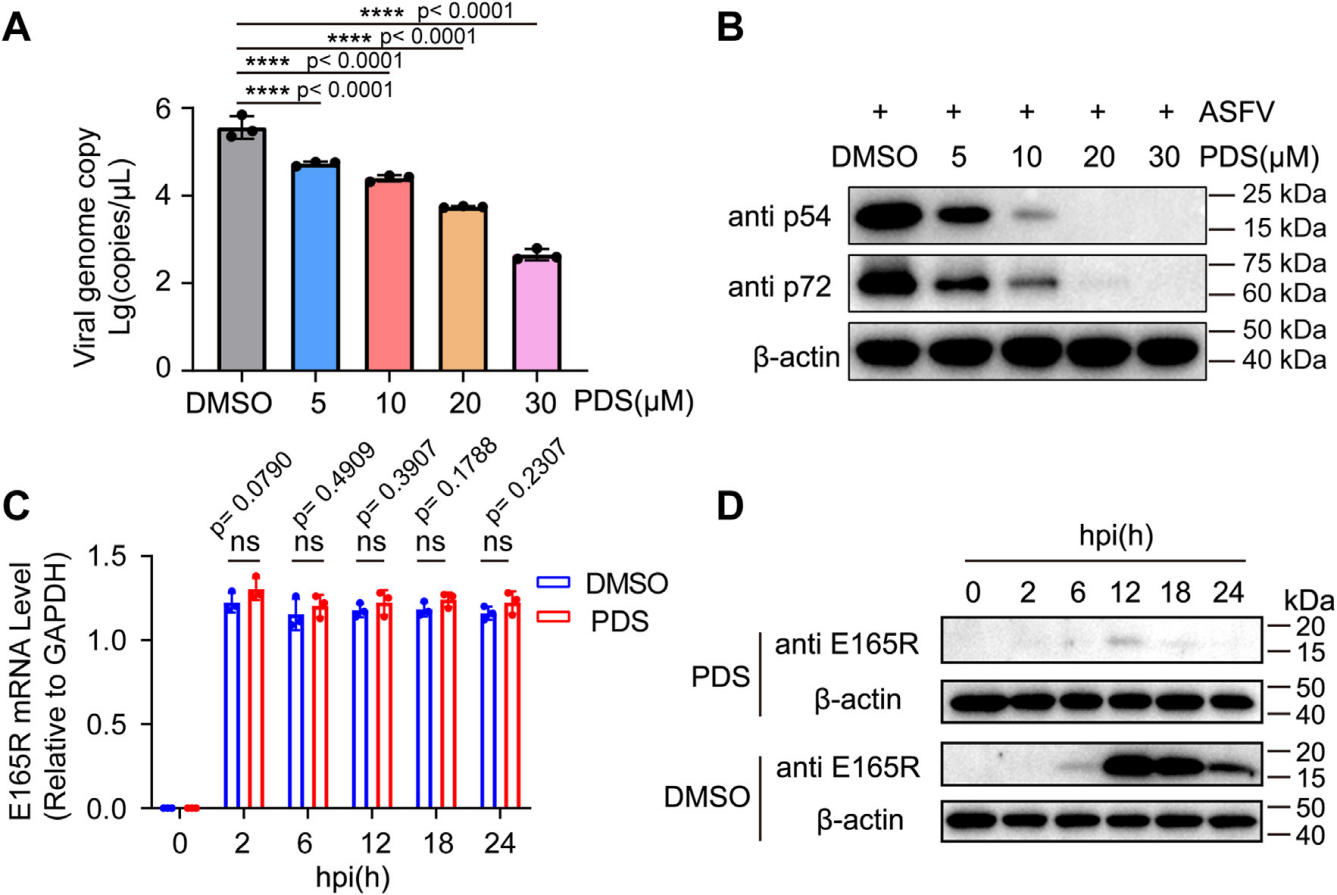
**PDS affects the genome copy number and protein synthesis of ASFV.** Vero cells were pretreated with different concentrations of PDS, infected with the ASFV at an MOI of 0.5, and then replaced with medium containing PDS (5 μM, 10 μM, 20 μM, and 30 μM). After 36 h of infection, the cells were harvested. *A*, Q-PCR determined the viral genome copy number, all experiments were performed in triplicate. Data were analyzed by One-way ANOVA with Dunnett's multiple comparisons test,∗∗∗∗*p* < 0.0001. *B*, Western blotting was used to assess the viral protein (p54 and p72) levels, and β-actin was used as a loading control. *C*, vero cells that were infected with ASFV at an MOI of 0.5 were treated with 20 μM PDS, at different hpi, the RNA was extracted and subjected to RT-qPCR analysis, while the proteins were harvested and subjected to Western blotting (*D*). The data represent the mean (±SD) of three independent experiments. Significant differences compared to the control are analyzed by unpaired two-tailed Student’s *t* test., ns, not significant. ASFV, African swine fever virus; MOI, multiplicity of infection; PDS, pyridostatin; q-PCR, quantitative PCR.

Upon infection of the cell by ASFV, the virus releases its viral genome, initiating viral genome replication and protein synthesis. Our previous findings have shown the presence of G4 structures in the ASFV genome, leading us to speculate that the G4-stabilizer PDS may disrupt ASFV genome replication and protein translation. To test this hypothesis, we treated Vero cells with varying concentrations of PDS and collected the cells at 36 h post infection to assess the genome copy number and protein levels of ASFV. The real-time quantitative PCR (RT-qPCR) results demonstrated that PDS addition could significantly decrease the ASFV genome copy, compared with the DMSO control sample, and the inhibitory effect displayed a concentration-dependent manner (Fig. 9*A*). Moreover, Western blot analysis revealed that PDS treatment dose dependently suppressed the synthesis of the p54 and p72 proteins of ASFV. Notably, 10 μM PDS significantly reduced the protein expression (Fig. 9*B*). Furthermore, RT-qPCR and Western blot experiments showed that PDS mainly affected E165R translation but not transcription, which was consistent with the findings of the ectopic assay (Fig. 7). These findings indicate that PDS can inhibit ASFV genome replication and viral protein expression.

Furthermore, to eliminate the possibility of PDS’s off-target effects, we treated ASFV-infected cells with varying concentrations of etoposide, a known DNA topoisomerase II (topo II) inhibitor. The immunofluorescence results demonstrated that ASFV was insensitive to etoposide. This finding indicates that the inhibitory effect of PDS on ASFV is attributable to its interaction with G4 rather than its role as a DNA topoisomerase II (topo II) inhibitor (Fig. S2).

## Discussions

ASFV, the only member of the Asfarviridae family and genus Asfivirus, has inflicted significant economic losses on the global pig industry. The genome of ASFV consists of double-stranded DNA encoding approximately 170 proteins. Due to the limited understanding of ASFV protein functions, effective antiviral strategies for control and prevention still need improvement. In this study, we identified a conserved PQS located on the sense strand of the E165R gene. This G4 motif exhibits characteristics of a canonical G4, comprising three tetrads and 1 to 7 nucleotide-long loops. Notably, the G4 stabilizers PDS and TMPyP4 effectively bind and stabilize both the DNA G-quadruplex (dG4) and the rG4 transcribed from the E165R gene. Functional assays, including EGFP reporter assays and Western blot analysis, demonstrated that PDS and TMPyP4 inhibit E165R expression in a G4-dependent manner. Moreover, treatment with PDS significantly suppressed ASFV proliferation in cells.

As noncanonical secondary structures, G4s have gained considerable attention due to their novel regulatory functions in biological processes (as reviewed in (41, 56, 57, 58)). Based on their loop properties, G4s can be classified into three categories: canonical G4s with loop lengths of 1 to 7 nucleotides, long-looped G4s with loops up to 30 nucleotides, and bulged G4s with incomplete G-tracts (59, 60). While long-looped G4s and bulged G4s have been identified in genomes and are biologically relevant, our study primarily focused on canonical G4s because previous studies have demonstrated an inverse correlation between G4 stability and loop length (42). Among the 119 PQSs obtained from QGRS analysis, the G4 motif located in the E165R gene was chosen as a candidate target due to its high score and the critical role played by the dUTPase encoded by E165R in ASFV replication.

For DNA viruses, PQS folds into G4s when double-stranded DNA unwinds into single-stranded DNA during replication and/or transcription. We speculate that the PQS located in the E165R gene potentially forms G4 DNA when the ASFV genome replicates in the nucleus. Previous studies have reported G4 formation in DNA viruses such as HSV, an alphaherpesvirus with a double-stranded DNA genome, which exhibits peak G4 formation during the virus replication stage (61). Previously, we reported G4 formation in pseudorabies virus, an HSV homolog, where G-rich sequences fold into G4s during replication (20, 21). In addition to DNA G4s, it has been shown that G-rich RNA sequences are prone to forming RNA G4s as there is no complementary strand to complete base pairing. Indeed, several RNA viruses, including HCV (62), Ebola virus (63), porcine epidemic diarrhea virus (64), and SARS-CoV-2 (40, 65), have been shown to form RNA G4s. Therefore, we speculated that the PQS in the E165R RNA of ASFV can also form an RNA G4 when transcribed. This speculation is partially supported by probing the RNA G4 structure formed by the synthesized RNA oligonucleotide of the E165R-G4 motif using BG4 (Fig. 5*E*).

To date, numerous ligands have been demonstrated to bind and stabilize G4 structures (66, 67, 68). Generally, these G4 binders interact with G4 structures primarily through π-π stacking and/or electrostatic interactions (69). Notably, PDS, a well-known G4 stabilizer, has been shown to bind and stabilize G4 structures formed in Epstein-Barr virus, human papillomavirus , HCV, and most recently, SARS-CoV-2. Similarly, this study found that PDS can bind and stabilize the G4 structure formed in the E165R gene, consequently suppressing protein synthesis and ASFV replication. It is important to note that although TMPyP4, another well-known G4 ligand, was able to inhibit E165R expression *in vitro*, ASFV-infected cells exhibited insensitivity to TMPyP4, indicating that the G4-mediated inhibitory effect is dependent on specific cellular contexts. Additionally, Muturi *et al.* reported the identification of two-tetrad G4 structures in the P1192R and D117L genes of ASFV, and the G4 stabilizer NMM was found to inhibit ASFV replication in porcine alveolar macrophages cells (70). This collective evidence from their work and ours suggests that G4 structures in the ASFV genome may serve as novel antiviral targets, albeit with different target sites. Although G4 stabilizers could interact with the G4 structure and serve as promising antiviral agents, the limitations of G4 stabilizers should also be considered when used as a medication or vaccine. Of note, the G4 ligands usually display limited bioavailability and unfavorable pharmacokinetics. Moreover, the G4 ligands that discriminate among G4 structures with different sequences or different topologies are still the main limitation (47).

Here, we revealed that G4 structures in ASFV potentially serve as antiviral targets, with G4 stabilizers being candidate drugs. However, it is important to highlight certain limitations that need to be further explored. First, although PDS, a G4 ligand used in this study, has been shown to inhibit ASFV replication in cells, further investigation is required to determine whether PDS exhibits similar inhibitory effects on ASFV replication *in vivo*. Second, bioinformatic analysis has identified many G2-tetrad forming motifs and noncanonical G4 types (long loop, bulged) in the ASFV genome; therefore, the selectivity of G4 ligands toward individual G4 sites and different G4 types needs to be considered for drug development and further exploration. Finally, G4 folding, unfolding, and regulatory functions are influenced by helicases and/or cellular factors. Previous studies have reported that helicases or cellular factors, such as BLM (71), FANCJ (72), and NCL (73), taking as examples, affect G4 formation and G4 biological functions. Thus, it is crucial to investigate which helicases participate in the G4-mediated regulatory role in the ASFV life cycle.

In conclusion, our study identified a conserved G4 structure with three tetrads formed in the coding region of E165R of ASFV. The G4 stabilizer PDS effectively binds and stabilizes this G4 structure, inhibiting E165R expression in a G4-dependent manner and subsequently inhibiting ASFV replication in cells. G4 structures may serve as potential drug targets against ASFV.

## Experimental procedures

### Reagents and antibodies

Q-PCR primers were obtained from Sangon Biotech, while other oligonucleotides and primers were synthesized by Genscript. PDS (C100489), Phen-DC3 (C103887), Braco-19 (C110527), and TMPyP4 (C108131) were purchased from ChemeGen, and NMM and TMPyP2 (T40846) were acquired from J&K Scientific. The anti-DDDDK tag antibody was procured from Abcam, the BG4 antibody was obtained from Sigma‒Aldrich, the horseradish peroxidase-conjugated secondary antibody was purchased from Jackson ImmunoResearch Laboratories, the etoposide was purchased from MedChemExpress, and the Alexa Fluor 555 conjugated goat anti-rabbit IgG (H + L) was sourced from Thermo Fisher Scientific.

### G4 sequence mapping

The reference genome of ASFV strain BA71V(NC_001659.2) and genomes of different ASFV strains were retrieved from the National Center for Biotechnology Information Genome database. The QGRS mapper was used to predict the putative GQ sequences on both strands of BA71V with four repeats of G groups and a minimum G group of 2. The maximum length was up to 35 nucleotides, with a loop size ranging from one to seven nucleotides. The algorithm pqsfinder was used to cross-check the putative GQ sequences on both strands of BA71V(https://pqsfinder.fi.muni.cz). Conservation analysis was performed on selected ASFV genomes by using the Jalview tool (74). LOGO representation of the conserved bases was obtained by WebLogo software online (https://weblogo.threeplusone.com/).

### CD spectrum measurements

In all, 10 μM DNA or RNA oligonucleotides were dissolved in a buffer containing 100 mM KCl and 25 mM Tris–HCl (pH 7.5) and slowly cooled from 95 °C to room temperature at a rate of 0.03 °C/s. Samples were then analyzed on Chirascan V100 (Applied Photophysics Ltd) at 25 °C with a bandwidth of 1 nm and a path length of 0.5 mm. CD spectra were recorded over a wavelength range of 200 to 320 nm three times, and the buffer spectrum was subtracted as the baseline.

### Gel mobility shift assay

The DNA labeled with 5′-FAM was dissolved in a buffer containing 100 mM KCl and 50 mM Tris–HCl (pH 7.5) at a concentration of 0.5 μM and gradually cooled from 95 °C to room temperature at a rate of 0.03 °C/s. Subsequently, the samples were electrophoresed at 100 V in a 15% native acrylamide gel at 4 °C for 4 h. Finally, the gels were imaged using the Amersham Imager 600 (GE HealthCare).

### Fluorescence turn-on assay

The oligonucleotides listed in Table S1 were annealed by cooling from 95 °C to room temperature at a rate of 0.03 °C/s. Subsequently, the annealed oligonucleotides were mixed with NMM or ThT at a 1:1 ratio at 25 °C for 1 h. The resulting samples were analyzed using the SpectraMax i3X multifunctional microplate reader. For the NMM-treated samples, the excitation wavelength was set to 393 nm, and the emission spectrum was collected in the range of 450 nm to 700 nm. In the case of the ThT-treated samples, the excitation wavelength was set to 442 nm, and the emission spectrum was collected from 450 nm to 700 nm.

### FRET melting experiments

For the FRET-melting experiments, the oligonucleotides were fluorescently labeled at the 5′ end with FAM and at the 3′ end with TAMRA. The oligonucleotides at a final concentration of 0.5 μM were annealed in buffer containing 100 mM KCl and 10 mM LiAsO4. Subsequently, the annealed oligonucleotides were mixed with different concentrations of PDS, TMPyP4, TMPyP2, PhenDC-3, or Braco-19, and incubated at 25 °C for 30 min. The sample was then subjected to a heating process from 25 to 95 °C at a rate of 0.01 °C/s using the QuantStudio 6 Flex Real-Time PCR System (Life Technologies), in which the FAM fluorescence was recorded.

### Taq polymerase termination experiment

For the Taq polymerase termination experiment, 0.5 μM single-stranded DNA (WT or mutant) was annealed with primers at a concentration of 0.5 μM in a buffer containing 100 mM KCl and 50 mM Tris–HCl. The annealed sample was then extended at 55 °C using 2 U of Taq DNA polymerase in the presence of various concentrations of PDS, TMPyP4, or TMPyP2 for 30 min. Following the extension reaction, the resulting extension products were collected by ethanol precipitation and subsequently analyzed using 20% denaturing PAGE containing 7 M urea.

### Cells and viruses

HEK293T cells (American Type Culture Collection, CRL-11268) and Vero cells were cultured in Dulbecco's modified Eagle's medium (Gibco) supplemented with 10% fetal bovine serum (FBS) (Gibco), 100 units/ml penicillin, and 100 mg/ml streptomycin sulfate (Sangon Biotech). The cells were maintained as monolayers in a 5% CO2 atmosphere at 37 °C. The recombinant ASFV with dural reporter genes (ASFV-Luc-EGFP) was obtained from our laboratory. They were propagated in Vero cells cultured in Dulbecco's modified Eagle's medium supplemented with 2% FBS. The virus stocks were divided and stored at −80 °C. All ASFV experiments and related samples were performed in the biosafety level-3 laboratory at Huazhong Agricultural University. Stringent safety measures were followed, and all waste generated during the experiments underwent appropriate sterilization and inactivation procedures to ensure safe disposal.

### Cell viability analysis

HEK93T and Vero cells were cultured in 96-well plates. Once the cells reached approximately 60% confluency, the growth medium was replaced with medium containing varying concentrations of PDS, TMPyP4, Baco19, or Phen-DC3. The cells were then allowed to grow for an additional 36 h. A CCK-8 kit (Biosharp) was used following the manufacturer's instructions to evaluate cell viability.

### Plasmids construction and transfection

The G4 sequence or its mutant counterpart was inserted downstream of the GFP gene start codon of the pEGFP-N1 plasmid. The resulting plasmids were named pEGFP-G4 and pEGFP-G4-Mut, respectively. The full-length coding sequences of ASFV E165R and its mutant variants were cloned into the p3 × FLAG-CMV-10 vector (Sigma‒Aldrich, E4901), yielding E165R-G4-WT and E165R-G4-Mut, respectively. All plasmids were validated by sequencing. Plasmid transfections were performed by using TurboFect reagent (Thermo Fisher Scientific) following the manufacturer's instructions.

### Flow cytometry assays

Upon reaching a density of 60%, HEK293T cells were subjected to medium replacement with varying concentrations of PDS, TMPyP4, or TMPyP2, followed by a 2-h incubation period. Subsequently, the cells were transfected with pEGFP-G4 and pEGFP-G4-Mut plasmids and incubated in medium supplemented with different concentrations of PDS, TMPyP4, or TMPyP2 for 24 h. To assess the proportion of GFP-positive cells, flow cytometry analysis was performed using CytoFLEX equipment (Beckman Coulter). The acquired data were processed and analyzed using CytExpert software provided with the CytoFLEX equipment.

### Western blotting assays

Vero or HEK293T cells subjected to specific treatments were harvested and lysed in radio immunoprecipitation assay lysis buffer supplemented with 1 mM phenylmethanesulfonyl fluoride (Beyotime) for 10 min. Protein quantification of the lysates was performed using the BCA protein quantification kit (Beijing Dingguo Changsheng Biotechnology Co, Ltd). The protein samples were then resolved on a 15% SDS‒PAGE gel and subsequently transferred onto a polyvinylidene fluoride membrane (Millipore). The membrane was blocked with 5% nonfat dry milk for 1 h, followed by overnight incubation at 4 °C with the indicated primary antibodies at a dilution of 1:1000. After washing, the membrane was incubated with a horseradish peroxidase-labeled secondary antibody at room temperature for 1 h. Finally, the GE AI600 imaging system was used for imaging using an enhanced chemiluminescence chemiluminescent substrate (Millipore).

### Immunofluorescence assays

Cells were seeded in 24-well plates with coverslips. When the cell density reached 60%, cells were transfected with FAM-labeled RNA (1 μg) and incubate for 24 h. The cells were then fixed in 4% paraformaldehyde in PBS for 30 min, followed by permeabilization in 0.1% Triton X-100 in PBS. The cells were then blocked with PBS containing 10% FBS for 1 h, followed by incubation with BG4 (Sigma‒Aldrich) diluted in PBS containing 10% FBS at 4 °C overnight. Alexa Fluor 568-conjugated goat anti-rabbit IgG (Invitrogen) was used as the secondary antibody, and incubated with the cells at room temperature for 1 h. The cells were then stained with DAPI (Invitrogen) for 12 min, and the coverslips were affixed to the slides and observed using a Zeiss LSM 800 confocal microscope.

### Virus titration

To determine the viral titer, ASFV-infected cells were subjected to three freeze‒thaw cycles at −80 °C. The resulting supernatant was collected and serially diluted 10-fold with cell culture medium. Subsequently, the 10-fold diluted viral suspension was added to 96-well plates containing a monolayer of cells, with each well receiving 100 μl of the viral suspension. Each dilution was performed in eight replicates. After 3 to 5 days of cell culture in the cell incubator, the cytopathic effects were observed and recorded. The TCID_50_ was calculated using the Reed-Muench method.

### Real-time quantitative PCR

To determine the gene expression levels of E165R, total RNA was extracted from the cells using TRIzol Reagent (Takara) and reverse transcribed into complementary DNA using a reverse transcription kit (Monad). Real-time PCR was then performed on the complementary DNA using SYBR Premix Ex Taq (Takara) with GAPDH as the reference gene. The primers used for E165R are listed in Table S4.

For quantification of the ASFV genome copy number, the experiment was conducted following the procedure recommended by the World Organization for Animal Health. Briefly, viral genomic DNA was extracted from Vero cells using the FastPure Viral DNA/RNA Mini Kit (Vazyme) following the manufacturer's protocols. The primer sequences are listed in Table S4.

### Statistical analysis

Statistical analysis was performed using GraphPad Prism 8 software (GraphPad Software, Inc, https://www.graphpad.com/), the One-way ANOVA, or the Student's *t* test where specified was used. Quantitative data were obtained from at least three independent experiments and are presented as the mean ± standard deviation. Statistical significance was determined at *p* < 0.05(∗), *p* < 0.01 (∗∗), *p* < 0.001 (∗∗∗), *p* < 0.0001 (∗∗∗∗), ns, not significant.

## Data availability

The data that support the findings of this study are available from the corresponding author upon reasonable request.

## Supporting information

This article contains supporting information.

## Conflict of interest

The authors declare that they have no conflict of interest with the contents of the article.

## Figures and Tables

**Figure S2:**