# Supplementary material for: Identification of a conserved G-quadruplex within the E165R of African swine fever virus (ASFV) as a potential antiviral target
Source: J Biol Chem. 2024 Jun 7;300(7):107453. doi: 10.1016/j.jbc.2024.107453 (PMC11261444; doi:10.1016/j.jbc.2024.107453)
Supplement: Supporting Tables [file mmc1.docx]

**Supplementary Table S1. Identification of potential quadruplex-forming sequences (PQS) within the sense strand(+) of ASFV genome**

| **Position** | **Length** | **PQS** | **G-core** |
| --- | --- | --- | --- |
| 10683 | 11 | GGGGGGGGGGG | 26 |
| 10933 | 11 | GGGGGGGGGGG | 26 |
| 11125 | 11 | GGGGGGGGGGG | 26 |
| 12758 | 11 | GGGGGGGGGGG | 26 |
| 12985 | 11 | GGGGGGGGGGG | 26 |
| 13114 | 11 | GGGGGGGGGGG | 26 |
| 17908 | 15 | GGCGGGAGAGGAAGG | 25 |
| 19928 | 27 | GGTTGTGTGGATAGAGGGAGTTCTAGG | 25 |
| 22127 | 15 | GGCAGGGGAGGAGGG | 25 |
| 23825 | 13 | GGAGAGGAGGGGG | 24 |
| 27459 | 22 | GGCTGTACTGGAAGGGACATGG | 22 |
| 28087 | 12 | GGCGGCAGGAGG | 25 |
| 28693 | 14 | GGTAGCGGAGGAGG | 23 |
| 35815 | 14 | GGAGGATTGGTAGG | 24 |
| 36006 | 20 | GGGACAGGCCCGGCACCAGG | 24 |
| 40121 | 17 | GGCGGTGAGGCTGCAGG | 22 |
| 40554 | 11 | GGCGGCGGTGG | 26 |
| 41680 | 27 | GGCAACTACGGCTGGCGGCCATGCAGG | 24 |
| 44477 | 21 | GGTCCACAGGCTCGGCTGTGG | 23 |
| 48682 | 20 | GGGCGGGAAGAAGGAGGAGG | 24 |
| 49789 | 19 | GGCGCTGGTGATAGGTCGG | 23 |
| 50762 | 19 | GGACACTAGGGCGGCAAGG | 22 |
| 53233 | 21 | GGCAACGGAACCAGAGGTGGG | 21 |
| 53411 | 11 | GGTGGCGGTGG | 26 |
| 55338 | 22 | GGAATGTGGCATGGAGATCCGG | 23 |
| 59295 | 11 | GGTGGTGGCGG | 26 |
| 59426 | 26 | GGTCCATGGCTCCTGTGGAGGCTTGG | 24 |
| 62099 | 22 | GGCTTCTGCGGGAACGGCCAGG | 22 |
| 62237 | 26 | GGCCTCCATGGGCATGGATAGATCGG | 23 |
| 65425 | 20 | GGCGGCCCTGGATCCCCTGG | 20 |
| 66823 | 17 | GGAGGAAAAGGCCGTGG | 23 |
| 73695 | 15 | GGGGGTTGGCAACGG | 23 |
| 73943 | 13 | GGCTTGGAGGTGG | 24 |
| 74087 | 23 | GGCTTAAGGGGCGGGGATATGGG | 26 |
| 74153 | 24 | GGCTGAAGGAAATGGTAAATCCGG | 23 |
| 74613 | 25 | GGTCGTGACGGCCTACGCGGAATGG | 22 |
| 75966 | 16 | GGCGGAGGCCAAGCGG | 21 |
| 77328 | 11 | GGTGGCGGAGG | 26 |
| 77661 | 20 | GGTTAGGACTCTTGGCTGGG | 23 |
| 77757 | 23 | GGGCAGGGCCGTGGATTTTCCGG | 23 |
| 77819 | 15 | GGGAGGTTGGAATGG | 25 |
| 78649 | 22 | GGTTGGGATCCGGAAACCTTGG | 22 |
| 79255 | 20 | GGTTAACGGTACTGAGGAGG | 21 |
| 80032 | 18 | GGAAGGCAATGAGGAAGG | 22 |
| 80603 | 17 | GGACCGGCCGGCTTTGG | 24 |
| 80672 | 17 | GGGGGAGAAATAGGAGG | 20 |
| 87908 | 20 | GGAATTTCGGGTTGGTATGG | 23 |
| 89336 | 23 | GGCCACGGCCGCATGGGATGTGG | 24 |
| 89525 | 21 | GGCGGTGAAAGGGTCCTCAGG | 21 |
| 92626 | 13 | GGACTGGAGGGGG | 24 |
| 93995 | 26 | GGTAGAAAGGCAATCCGGTTCACCGG | 26 |
| 94185 | 11 | GGGGGTGGCGG | 26 |
| 94297 | 14 | GGCTGGCGGCATGG | 24 |
| 94343 | 21 | GGCCGTGGGTGCTGGCATAGG | 25 |
| 95241 | 22 | GGTGTTTGGAAACCACGGTGGG | 21 |
| 95297 | 27 | GGAATATCGGGAGTAGCCGGAAATAGG | 24 |
| 100228 | 14 | GGGAGGCGGGGTGG | 26 |
| 100244 | 14 | GGAGGAATGGGTGG | 24 |
| 103711 | 15 | GGCATCGGTGGGTGG | 23 |
| 104701 | 13 | GGGGGTAGGTTGG | 25 |
| 105157 | 16 | GGCGAGGCGAGGAAGG | 25 |
| 107461 | 17 | GGCGGCCCCATCGGTGG | 20 |
| 107581 | 21 | GGAACCGGCTCGAGGATCGGG | 25 |
| 109026 | 21 | GGTCTGGATCATGGAGTCAGG | 24 |
| 109086 | 13 | GGAGGGTGGGGGG | 25 |
| 109390 | 20 | GGTCCTACCGGATGGAATGG | 21 |
| 109497 | 17 | GGCTACTAAGGAGGTGG | 20 |
| 112031 | 17 | GGTTCAAGTGGTGGAGG | 20 |
| 113409 | 19 | GGCCACGGACCGGTGGTGG | 25 |
| 115767 | 20 | GGTTGCGGTGGTGGAAACGG | 26 |
| 119640 | 18 | GGGAGGATAGGCAGCAGG | 23 |
| 119743 | 24 | GGAACTGGCAAAGGGCCCTTCTGG | 23 |
| 119882 | 19 | GGAATAGGAAAAGGGCAGG | 25 |
| 120217 | 16 | GGGGGGAATGGTGTGG | 25 |
| 122596 | 23 | GGCGAGGACTTAAGGAGATTCGG | 23 |
| 123809 | 14 | GGATAGGTGGGCGG | 24 |
| 124644 | 26 | GGCCTCAAAGGTATTGAGGACGGTGG | 24 |
| 124684 | 22 | GGCGTGATGGATGGACTAAGGG | 22 |
| 125057 | 21 | GGCCTTCCAGGTTGGACGAGG | 21 |
| 125882 | 17 | GGCGCTGGAGGGGCTGG | 24 |
| 125991 | 24 | GGCCCTGGGGGCCTGGTGAGGTGG | 25 |
| 127200 | 25 | GGAATGTGGGACTGCAGGGAGGTGG | 25 |
| 127252 | 22 | GGAAATGGTGGTGAGGCTCAGG | 24 |
| 127293 | 14 | GGAGGAGGTTTAGG | 23 |
| 128004 | 14 | GGAGGAGGCGCAGG | 23 |
| 128082 | 26 | GGTGGTCTGGCTGGGAGGAAGACCGG | 26 |
| 128624 | 23 | GGAGCATGGACTCTGGAGGGTGG | 26 |
| 128737 | 19 | GGTGAAAGGGCAAGGCCGG | 23 |
| 130246 | 23 | GGATATGGTAAAGGTATATAAGG | 23 |
| 131141 | 21 | GGGGGGAACCAACGGCGTCGG | 21 |
| 131809 | 18 | GGACCGGCCAGCGGAAGG | 23 |
| 131993 | 20 | GGGAGGGAAAAGGGCGCAGG | 24 |
| 133077 | 25 | GGCTCCAGGCACCTGGGAGGAAAGG | 25 |
| 133202 | 17 | GGACGGCCAGCGGGCGG | 23 |
| 134756 | 24 | GGCGGTGGTGACGCGGGTCTGAGG | 24 |
| 136701 | 14 | GGTGGAAGGCCAGG | 24 |
| 138195 | 14 | GGGGGCCGGGATGG | 24 |
| 138318 | 11 | GGAGGGGGTGG | 26 |
| 140318 | 16 | GGTTGGCAGGGATTGG | 25 |
| 141263 | 16 | GGCAGGCAGAGGGCGG | 24 |
| 142203 | 15 | GGACGGGGAAGGAGG | 24 |
| 142908 | 18 | GGAAAAGGTTGTGGTGGG | 24 |
| 142983 | 20 | GGGCCGGTCCAGGGGTGTGG | 24 |
| 144283 | 25 | GGCTTCTTGGGGGTCGGTGTCTTGG | 25 |
| 144352 | 17 | GGAGGGGGGCTGAGAGG | 21 |
| 145545 | 21 | GGCCGCAGGTGCGGCCGCTGG | 24 |
| 145693 | 23 | GGTTTAGAGGTACCTGGTTGTGG | 24 |
| 146678 | 23 | GGAGGATCTGAAGGACAAAACGG | 20 |
| 147133 | 24 | GGCTTCAGGGAGCCATGGAGGCGG | 24 |
| 149688 | 15 | GGAAGGACCTGGGGG | 23 |
| 149962 | 21 | GGTGCAGTTGGTGGCCCAAGG | 20 |
| 150061 | 31 | GGGTAGATTTGGGAGCACGGGCGAGGCCGGG | 50 |
| 150524 | 18 | GGTGGTGAAGGGGAATGG | 22 |
| 153293 | 17 | GGTGTCGGTTGGCAAGG | 24 |
| 156230 | 18 | GGTAGGTTGGGGTGTTGG | 24 |
| 157069 | 20 | GGGTATTGGTGGAACATTGG | 21 |
| 157421 | 28 | GGTGCATATGGGTATTCTGGAGGAAAGG | 25 |
| 159829 | 19 | GGTCTTTGGGCTGTGGTGG | 22 |
| 165833 | 19 | GGTTTATGGTGGCATGAGG | 22 |

**Supplementary Table S2. Identification of potential quadruplex-forming sequences (PQS) within the anti-sense strand(-) of ASFV genome**

| **Position** | **Length** | **QGRS** | **G-Score** |
| --- | --- | --- | --- |
| 2306 | 14 | GGCTGGGCGGCGGG | 26 |
| 5380 | 11 | GGGGGCGGCGG | 26 |
| 5475 | 22 | GGTCCCTGGGAGCAGGTGGCGG | 25 |
| 6119 | 14 | GGTTAAGGAGGGGG | 23 |
| 9999 | 18 | GGCCCTTTGGTGGTTTGG | 21 |
| 11057 | 11 | GGAGGAGGAGG | 26 |
| 13479 | 21 | GGAAGTGGCTTAACAGGTAGG | 21 |
| 16899 | 23 | GGGAGGGCCCGAGGGTGAATGGG | 48 |
| 18154 | 18 | GGCTTGGTGGGATCACGG | 23 |
| 19033 | 17 | GGTAGGGCTGGGAATGG | 26 |
| 20103 | 23 | GGTCAGGCACATGGTCACCTTGG | 22 |
| 21950 | 18 | GGAATGGTCGAGGTCAGG | 25 |
| 22468 | 17 | GGTAATCTCGGTGGGGG | 20 |
| 23477 | 26 | GGTGCATAAGGGAAAGGTTGTGTCGG | 23 |
| 24524 | 20 | GGCAACTGGCGGGCCAGCGG | 23 |
| 24876 | 19 | GGCCCAAGAGGAGGAATGG | 20 |
| 27110 | 20 | GGACCGGCCCGATGGTATGG | 23 |
| 28044 | 15 | GGATAGGCCAGGGGG | 24 |
| 30592 | 21 | GGACGATAGGTATGGTCGCGG | 23 |
| 31033 | 19 | GGTTGTGGTTTTGGATAGG | 25 |
| 33217 | 15 | GGCCGGGGGTTATGG | 23 |
| 34425 | 14 | GGCGGCCCGGAGGG | 24 |
| 37051 | 23 | GGATAGAGGTGGGAGGAGCCCGG | 26 |
| 37998 | 20 | GGAGGGCCCGCTTGGGTTGG | 21 |
| 38817 | 21 | GGAGGGTGTAATGGTAGGAGG | 22 |
| 38892 | 17 | GGCGGTGGTAAGAATGG | 20 |
| 40060 | 20 | GGCCTTCACGGCGGGGTGGG | 21 |
| 40308 | 14 | GGTCGGAGGCGTGG | 24 |
| 41660 | 19 | GGTTGGCTACTTTGGATGG | 21 |
| 44257 | 27 | GGAAAAAGGACGCCCTGGCTCTTGTGG | 24 |
| 44767 | 14 | GGCAAGGCCGGGGG | 24 |
| 45067 | 22 | GGTCCAAGGTGATTGTGGAGGG | 21 |
| 45158 | 17 | GGTATTTGGGAGGGTGG | 23 |
| 45311 | 14 | GGAGGGGGATAAGG | 23 |
| 45455 | 26 | GGCCTATGGATACGGGGAGCAAGAGG | 24 |
| 46239 | 25 | GGCTATATGGAAAGCAAGGGAAAGG | 23 |
| 47371 | 21 | GGAAAGGGCCCAAGGGGAAGG | 25 |
| 51315 | 11 | GGGGGAGGAGG | 26 |
| 51406 | 14 | GGCCTGGTGGTAGG | 24 |
| 51912 | 25 | GGATGCTGTGGGAGATGGTGCTAGG | 24 |
| 52357 | 19 | GGAAGAAGGGGCGGTGCGG | 24 |
| 52978 | 23 | GGATGAGGCCCTTAGGCTTAAGG | 24 |
| 53150 | 23 | GGAAAAAAGGGAAAGGACTTAGG | 24 |
| 53563 | 18 | GGCAAGGCGTTATGGAGG | 21 |
| 54338 | 21 | GGGAGGCACGGGAAAGCGTGG | 21 |
| 54688 | 13 | GGCTGGAGGTAGG | 25 |
| 55313 | 24 | GGCCGTCGGAGCGGGCTCCTCCGG | 23 |
| 55475 | 17 | GGAGGCACATCAGGAGG | 20 |
| 55694 | 20 | GGTGGCCACTGGCGCCAAGG | 21 |
| 55835 | 18 | GGAGGCGCAGGCCTACGG | 22 |
| 56607 | 13 | GGTGAGGCGGTGG | 24 |
| 56683 | 16 | GGTCCGTGGCCGGGGG | 22 |
| 56951 | 23 | GGAATGGATGACGGATTTTCTGG | 22 |
| 57518 | 17 | GGTTATGGGGGACAAGG | 23 |
| 59371 | 17 | GGTACTGGGAGGAATGG | 24 |
| 60054 | 25 | GGAACTAGGTCTGTAAGGCTACTGG | 24 |
| 60542 | 23 | GGTATAGGAATGTACGGTTTCGG | 23 |
| 61262 | 29 | GGTCTTTATGGCCTGTGAGGGGTCATTGG | 26 |
| 63725 | 13 | GGAGGCGGGGAGG | 25 |
| 64518 | 24 | GGGGAGAGGAGCTGCGGCAAATGG | 25 |
| 66413 | 20 | GGTGAATTAGGCCTGGAGGG | 21 |
| 67349 | 26 | GGTCAGGCGGCCCTGCGGGGAATTGG | 26 |
| 68606 | 20 | GGCCCGGGTTTGGCCGCAGG | 24 |
| 70791 | 17 | GGTAGGATTTGGCTCGG | 24 |
| 71265 | 20 | GGCGATGGTCCTCAGGGCGG | 22 |
| 72192 | 12 | GGCCGGTGGTGG | 25 |
| 74041 | 17 | GGAGGATAGTGGCGAGG | 22 |
| 78613 | 15 | GGTAGGCACTGGTGG | 23 |
| 81165 | 20 | GGCCAAAGGGTCGCCGGAGG | 21 |
| 81737 | 20 | GGGCACGGCCCAGATGGGGG | 20 |
| 82049 | 14 | GGTCGGCCAGGAGG | 24 |
| 82067 | 18 | GGTGGAGGGAACTAGTGG | 20 |
| 86732 | 19 | GGAGGCCATCTGGCAGCGG | 21 |
| 91569 | 21 | GGGCTGGGTTTTGGGCACAGG | 25 |
| 94445 | 23 | GGGAGGCGTCGAGGACGAAGAGG | 21 |
| 95749 | 23 | GGTTACCGGGGTCGGGGTTATGG | 26 |
| 95852 | 20 | GGACGCGGCGACCATGGTGG | 20 |
| 96365 | 20 | GGCCGGGATGGTCTCAATGG | 21 |
| 97061 | 20 | GGCGGATGTATTGGCCAAGG | 20 |
| 97432 | 26 | GGAGCGAGGGAACAGAGGCGGAGTGG | 26 |
| 97722 | 17 | GGGGGTTCCAGCGGGGG | 20 |
| 98536 | 23 | GGAATATGTGGAAACGGAAGAGG | 23 |
| 98578 | 20 | GGAGGAGTCCGAGGACAAGG | 20 |
| 100090 | 26 | GGAGATTGAGGCGGCTTTGGAGGTGG | 23 |
| 101736 | 16 | GGGGGCAAAGGGTGGG | 23 |
| 102544 | 18 | GGCGGACGGTTTTAAAGG | 20 |
| 103750 | 23 | GGTTGGGTTGAAATGGTAGTAGG | 22 |
| 104640 | 23 | GGTTCAGGATAATGGTTTCCAGG | 24 |
| 105241 | 26 | GGAAGTAGGCATTGCAGGTGCTTTGG | 24 |
| 107243 | 19 | GGCGCAGGCGGAGGAATGG | 25 |
| 107364 | 19 | GGTGCACATGGTGGGAGGG | 21 |
| 108266 | 20 | GGAGCGGCGGGCATAGCTGG | 21 |
| 108502 | 21 | GGCTATTTGGAGGTGCTTTGG | 21 |
| 109925 | 14 | GGTGGGGAGGACGG | 26 |
| 110739 | 22 | GGCCATAGGAGCCATGGAGGGG | 23 |
| 110952 | 17 | GGAAAAGGAGGATTTGG | 23 |
| 111877 | 21 | GGGGAATGGCCGATTGGAGGG | 22 |
| 112045 | 15 | GGTGAGGGAGGTGGG | 25 |
| 112703 | 29 | GGGATATTGGGTAGTAACGGGATACTAGG | 26 |
| 112753 | 21 | GGATTCAGGTGAAGGACATGG | 25 |
| 112777 | 21 | GGATGGAGGACATGGTTTAGG | 25 |
| 112799 | 29 | GGAGGACATGGTTTAGGTGGAGGACATGG | 26 |
| 112832 | 20 | GGTGGAGGACATGGTTTAGG | 26 |
| 112853 | 20 | GGACGACACGGTTTGGGTGG | 21 |
| 112880 | 29 | GGTTTAGGTGGAGGACATGGTTTGGGTGG | 26 |
| 112911 | 16 | GGCATGGCTTGGGTGG | 25 |
| 112928 | 20 | GGACATGGTTTAGGTGGCGG | 26 |
| 112952 | 20 | GGTTTAGGTAGGGGAAATGG | 26 |
| 113042 | 26 | GGAAGTAATGGTTCTCTGGGAGATGG | 24 |
| 115718 | 14 | GGAGGTAGGTTTGG | 24 |
| 116125 | 15 | GGTAAAGGGAGGAGG | 23 |
| 116503 | 25 | GGTGTAATGGGTGTGCCTGGTAAGG | 22 |
| 117409 | 13 | GGTCGGCAGGAGG | 25 |
| 118970 | 17 | GGTTGGACGTCGGTAGG | 23 |
| 119391 | 22 | GGGTAGGCAGACGGGGGGTTGG | 23 |
| 119751 | 20 | GGGAACGGTGGTCAAAAAGG | 20 |
| 119984 | 24 | GGAACAAGGGCATTGCGGCGCTGG | 24 |
| 120423 | 29 | GGGAGAAATGGAGCATTGGGTGCTTACGG | 26 |
| 120749 | 23 | GGCTTTCCGGGATAAAAAGGCGG | 20 |
| 123218 | 17 | GGTGTGGCCTCTGGAGG | 22 |
| 124328 | 14 | GGAGTGGGAGGCGG | 24 |
| 126371 | 17 | GGAAACAGGTAGGCCGG | 23 |
| 127558 | 23 | GGGGGTGTGCAAGGTTAACGAGG | 20 |
| 128352 | 19 | GGTTGCGGTGGGCATAAGG | 23 |
| 129393 | 25 | GGCCCCAGGGCGTAGGCCTCCCTGG | 24 |
| 130341 | 15 | GGACGGCGCAGGAGG | 23 |
| 130663 | 17 | GGTGCGGCAGTGGATGG | 24 |
| 130700 | 17 | GGTGAGCGGCTGGTGGG | 23 |
| 130921 | 17 | GGAGGCTGTGGAGTTGG | 23 |
| 132457 | 17 | GGCGGTGTGGAATTCGG | 22 |
| 133285 | 22 | GGCGGTCAAGAGGAGTTTTTGG | 20 |
| 133497 | 26 | GGCACACAGGAAACTTGGCCGCTAGG | 26 |
| 133961 | 26 | GGCTATCAGGAGCTGCGGCGAAGTGG | 26 |
| 134337 | 26 | GGATTTGCGGTGAATGGGAGACACGG | 26 |
| 135114 | 21 | GGATCGGGGCCCACGGATCGG | 24 |
| 135545 | 16 | GGATGGATAGGATTGG | 25 |
| 135726 | 22 | GGAGAAAGGACAGGGTTAATGG | 25 |
| 136366 | 27 | GGTGTTGGGCTATTTCGGAAAAAATGG | 24 |
| 139716 | 22 | GGTTATGGCCCTGGCGAGCAGG | 24 |
| 141473 | 21 | GGCTTCATGGTATTGGTTAGG | 23 |
| 150077 | 23 | GGCCCAGGGAATACAGGTGCAGG | 24 |
| 151606 | 17 | GGCTTGGGTCAGGACGG | 24 |
| 152203 | 19 | GGCCTCGTTGGTGGAAAGG | 20 |
| 152654 | 23 | GGAGAAGGTTCGTATGGACATGG | 23 |
| 159827 | 18 | GGAAGGAGTGGAAAAAGG | 23 |
| 160109 | 28 | GGAATTCTTGGCCTTCTGGCCAGCCAGG | 25 |
| 160689 | 28 | GGAATTCTTGGCCTTCTGGCCAACCAGG | 25 |
| 161115 | 28 | GGAATTCTTGGCCTGCTGGCCAATCAGG | 25 |
| 165737 | 17 | GGTTTGTGGTGGCATGG | 22 |

**Supplementary Table S3. Identification of potential quadruplex-forming sequences (PQS) within the both strands of ASFV genome by using pqsfinder.**

| **start** | **end** | **width** | | | **sequence** |  | **strand** | | | **score** | |
| --- | --- | --- | --- | --- | --- | --- | --- | --- | --- | --- | --- |
| 4349 | 4365 | | 17 | CCATGCCACCACAAACC | | | | - | 24 | |  |
| 10258 | 10275 | | 18 | CCTTTTTCCACTCCTTCC | | | | - | 23 | |  |
| 10683 | 10693 | | 11 | GGGGGGGGGGG | | | | + | 33 | |  |
| 10930 | 10940 | | 11 | GGGGGGGGGGG | | | | + | 33 | |  |
| 11125 | 11135 | | 11 | GGGGGGGGGGG | | | | + | 33 | |  |
| 12756 | 12766 | | 11 | GGGGGGGGGGG | | | | + | 33 | |  |
| 12985 | 12995 | | 11 | GGGGGGGGGGG | | | | + | 33 | |  |
| 13114 | 13124 | | 11 | GGGGGGGGGGG | | | | + | 33 | |  |
| 17881 | 17899 | | 19 | CCTTTCCACCAACGAGGCC | | | | - | 21 | |  |
| 17908 | 17922 | | 15 | GGCGGGAGAGGAAGG | | | | + | 27 | |  |
| 18480 | 18496 | | 17 | CCGTCCTGACCCAAGCC | | | | - | 24 | |  |
| 22127 | 22140 | | 14 | GGCAGGGGAGGAGG | | | | + | 29 | |  |
| 23825 | 23837 | | 13 | GGAGAGGAGGGGG | | | | + | 30 | |  |
| 28087 | 28098 | | 12 | GGCGGCAGGAGG | | | | + | 32 | |  |
| 28693 | 28706 | | 14 | GGTAGCGGAGGAGG | | | | + | 29 | |  |
| 34547 | 34560 | | 14 | CCTATCCATCCACC | | | | - | 29 | |  |
| 35815 | 35828 | | 14 | GGAGGATTGGTAGG | | | | + | 29 | |  |
| 36007 | 36025 | | 19 | GGACAGGCCCGGCACCAGG | | | | + | 21 | |  |
| 36141 | 36157 | | 17 | CCATATTGGCCGCCTCC | | | | - | 24 | |  |
| 37629 | 37645 | | 17 | CCGAATTCCACACCGCC | | | | - | 24 | |  |
| 39165 | 39181 | | 17 | CCAACTCCACAGCCTCC | | | | - | 24 | |  |
| 39387 | 39402 | | 16 | CCACCAGCCGCTCACC | | | | - | 26 | |  |
| 39423 | 39439 | | 17 | CCATCCACTGCCGCACC | | | | - | 24 | |  |
| 39747 | 39761 | | 15 | CCTCCTGCGCCGTCC | | | | - | 27 | |  |
| 40121 | 40137 | | 17 | GGCGGTGAGGCTGCAGG | | | | + | 24 | |  |
| 40551 | 40561 | | 11 | GGCGGCGGCGG | | | | + | 33 | |  |
| 41677 | 41694 | | 18 | GGTGGCAACTACGGCTGG | | | | + | 23 | |  |
| 41740 | 41753 | | 14 | CCACCGCAACCTCC | | | | - | 29 | |  |
| 43715 | 43731 | | 17 | CCGGCCTACCTGTTTCC | | | | - | 24 | |  |
| 44485 | 44500 | | 16 | GGCTCGGCTGTGGAGG | | | | + | 26 | |  |
| 45761 | 45774 | | 14 | CCGCCTCCCACTCC | | | | - | 29 | |  |
| 46868 | 46884 | | 17 | CCTCCAGAGGCCACACC | | | | - | 24 | |  |
| 48687 | 48701 | | 15 | GGAAGAAGGAGGAGG | | | | + | 27 | |  |
| 49789 | 49807 | | 19 | GGCGCTGGTGATAGGTCGG | | | | + | 21 | |  |
| 50332 | 50350 | | 19 | CCTTTTTGACCACCGTTCC | | | | - | 21 | |  |
| 50695 | 50710 | | 16 | CCCCCGTCTGCCTACC | | | | - | 26 | |  |
| 50759 | 50775 | | 17 | GGGGGACACTAGGGCGG | | | | + | 24 | |  |
| 51116 | 51132 | | 17 | CCTACCGACGTCCAACC | | | | - | 24 | |  |
| 52681 | 52693 | | 13 | CCTCCTGCCGACC | | | | - | 30 | |  |
| 53233 | 53252 | | 20 | GGCAACGGAACCAGAGGTGG | | | | + | 20 | |  |
| 53411 | 53421 | | 11 | GGTGGCGGTGG | | | | + | 33 | |  |
| 53963 | 53977 | | 15 | CCTCCTCCCTTTACC | | | | - | 27 | |  |
| 54371 | 54384 | | 14 | CCAAACCTACCTCC | | | | - | 29 | |  |
| 57131 | 57150 | | 20 | CCATTTCCCCTACCTAAACC | | | | - | 20 | |  |
| 57155 | 57168 | | 14 | CCGCCACCTAAACC | | | | - | 29 | |  |
| 57173 | 57186 | | 14 | CCTCCACCCAAGCC | | | | - | 29 | |  |
| 57191 | 57204 | | 14 | CCGCCACCCAAACC | | | | - | 29 | |  |
| 57209 | 57222 | | 14 | CCTCCACCTAAACC | | | | - | 29 | |  |
| 57234 | 57252 | | 19 | CCAAACCGTGTCGTCCACC | | | | - | 21 | |  |
| 57263 | 57276 | | 14 | CCTCCACCTAAACC | | | | - | 29 | |  |
| 57281 | 57294 | | 14 | CCTCCACCTAAACC | | | | - | 29 | |  |
| 57299 | 57312 | | 14 | CCTCCACCTAAACC | | | | - | 29 | |  |
| 57317 | 57330 | | 14 | CCTCCATCCAAACC | | | | - | 29 | |  |
| 57356 | 57372 | | 17 | CCACCCAAACCACTACC | | | | - | 24 | |  |
| 58047 | 58060 | | 14 | CCTCCCTCACCCCC | | | | - | 29 | |  |
| 58206 | 58223 | | 18 | CCTCCAATCGGCCATTCC | | | | - | 23 | |  |
| 59134 | 59150 | | 17 | CCAAATCCTCCTTTTCC | | | | - | 24 | |  |
| 59295 | 59305 | | 11 | GGTGGTGGCGG | | | | + | 33 | |  |
| 59344 | 59363 | | 20 | CCTCCATGGCTCCTATGGCC | | | | - | 20 | |  |
| 59433 | 59451 | | 19 | GGCTCCTGTGGAGGCTTGG | | | | + | 21 | |  |
| 60164 | 60177 | | 14 | CCGTCCTCCCCACC | | | | - | 29 | |  |
| 61817 | 61836 | | 20 | CCAGCTATGCCCGCCGCTCC | | | | - | 20 | |  |
| 62096 | 62115 | | 20 | GGGGGCTTCTGCGGGAACGG | | | | + | 20 | |  |
| 62721 | 62738 | | 18 | CCTCCCACCATGTGCACC | | | | - | 23 | |  |
| 62841 | 62853 | | 13 | CCATTCCTCCGCC | | | | - | 30 | |  |
| 65425 | 65444 | | 20 | GGCGGCCCTGGATCCCCTGG | | | | + | 20 | |  |
| 66823 | 66839 | | 17 | GGAGGAAAAGGCCGTGG | | | | + | 24 | |  |
| 67541 | 67558 | | 18 | CCTTTAAAACCGTCCGCC | | | | - | 23 | |  |
| 68352 | 68366 | | 15 | CCACCCTTTGCCCCC | | | | - | 27 | |  |
| 69990 | 70003 | | 14 | CCTCCAAAGCCGCC | | | | - | 29 | |  |
| 71505 | 71524 | | 20 | CCTTGTCCTCGGACTCCTCC | | | | - | 20 | |  |
| 71550 | 71569 | | 20 | CCGTTTCCACATATTCCTCC | | | | - | 20 | |  |
| 72364 | 72380 | | 17 | CCCCCGCTGGAACCCCC | | | | - | 24 | |  |
| 72642 | 72654 | | 13 | CCCCCACTCCGCC | | | | - | 30 | |  |
| 73022 | 73041 | | 20 | CCTTGGCCAATACATCCGCC | | | | - | 20 | |  |
| 73695 | 73709 | | 15 | GGGGGTTGGCAACGG | | | | + | 27 | |  |
| 73718 | 73737 | | 20 | CCATTGAGACCATCCCGGCC | | | | - | 20 | |  |
| 73943 | 73955 | | 13 | GGCTTGGAGGTGG | | | | + | 30 | |  |
| 74231 | 74250 | | 20 | CCACCATGGTCGCCGCGTCC | | | | - | 20 | |  |
| 75966 | 75981 | | 16 | GGCGGAGGCCAAGCGG | | | | + | 26 | |  |
| 77328 | 77338 | | 11 | GGTGGCGGAGG | | | | + | 33 | |  |
| 77661 | 77679 | | 19 | GGTTAGGACTCTTGGCTGG | | | | + | 21 | |  |
| 77820 | 77833 | | 14 | GGAGGTTGGAATGG | | | | + | 29 | |  |
| 78513 | 78532 | | 20 | CCTGTGCCCAAAACCCAGCC | | | | - | 20 | |  |
| 79255 | 79274 | | 20 | GGTTAACGGTACTGAGGAGG | | | | + | 20 | |  |
| 80032 | 80049 | | 18 | GGAAGGCAATGAGGAAGG | | | | + | 23 | |  |
| 80593 | 80609 | | 17 | GGCCGGCTTTGGACCGG | | | | + | 24 | |  |
| 80612 | 80628 | | 17 | GGCTTTGGACCGGCAGG | | | | + | 24 | |  |
| 80672 | 80688 | | 17 | GGGGGAGAAATAGGAGG | | | | + | 24 | |  |
| 83352 | 83370 | | 19 | CCGCTGCCAGATGGCCTCC | | | | - | 21 | |  |
| 87908 | 87927 | | 20 | GGAATTTCGGGTTGGTATGG | | | | + | 20 | |  |
| 88018 | 88035 | | 18 | CCACTAGTTCCCTCCACC | | | | - | 23 | |  |
| 88040 | 88053 | | 14 | CCTCCTGGCCGACC | | | | - | 29 | |  |
| 88346 | 88364 | | 19 | CCCCCATCTGGGCCGTGCC | | | | - | 21 | |  |
| 88918 | 88937 | | 20 | CCTCCGGCGACCCTTTGGCC | | | | - | 20 | |  |
| 91475 | 91489 | | 15 | CCACCAGTGCCTACC | | | | - | 27 | |  |
| 92626 | 92638 | | 13 | GGACTGGAGGGGG | | | | + | 30 | |  |
| 94185 | 94195 | | 11 | GGGGGTGGCGG | | | | + | 33 | |  |
| 94297 | 94310 | | 14 | GGCTGGCGGCATGG | | | | + | 29 | |  |
| 94340 | 94357 | | 18 | GGTGGCCGTGGGTGCTGG | | | | + | 23 | |  |
| 96045 | 96061 | | 17 | CCTCGCCACTATCCTCC | | | | - | 24 | |  |
| 97899 | 97910 | | 12 | CCACCACCGGCC | | | | - | 32 | |  |
| 98822 | 98840 | | 19 | CCTGAGGACCATCGCCCCC | | | | - | 21 | |  |
| 99295 | 99311 | | 17 | CCGAGCCAAATCCTACC | | | | - | 24 | |  |
| 100225 | 100236 | | 12 | GGAGGGAGGCGG | | | | + | 32 | |  |
| 100237 | 100248 | | 12 | GGTGGGTGGAGG | | | | + | 32 | |  |
| 101477 | 101496 | | 20 | CCTGCGGCCAAACCCGGGCC | | | | - | 20 | |  |
| 102736 | 102753 | | 18 | CCGCAGGGCCGCCTGACC | | | | - | 23 | |  |
| 103674 | 103692 | | 19 | CCAGGCCTAATTCACCGCC | | | | - | 21 | |  |
| 103711 | 103725 | | 15 | GGCATCGGTGGGTGG | | | | + | 27 | |  |
| 104701 | 104713 | | 13 | GGGGGTAGGTTGG | | | | + | 30 | |  |
| 105162 | 105175 | | 14 | GGCGAGGAAGGCGG | | | | + | 29 | |  |
| 105568 | 105585 | | 18 | CCGCAGCTCCTCTCCCCC | | | | - | 23 | |  |
| 106365 | 106377 | | 13 | CCTCCCCGCCTCC | | | | - | 30 | |  |
| 107461 | 107477 | | 17 | GGCGGCCCCATCGGTGG | | | | + | 24 | |  |
| 107578 | 107595 | | 18 | GGTGGAACCGGCTCGAGG | | | | + | 23 | |  |
| 109086 | 109097 | | 12 | GGAGGGTGGGGG | | | | + | 32 | |  |
| 109390 | 109409 | | 20 | GGTCCTACCGGATGGAATGG | | | | + | 20 | |  |
| 109497 | 109513 | | 17 | GGCTACTAAGGAGGTGG | | | | + | 24 | |  |
| 110715 | 110731 | | 17 | CCATTCCTCCCAGTACC | | | | - | 24 | |  |
| 112031 | 112047 | | 17 | GGTTCAAGTGGTGGAGG | | | | + | 24 | |  |
| 112568 | 112584 | | 17 | CCTTGTCCCCCATAACC | | | | - | 24 | |  |
| 113404 | 113419 | | 16 | CCCCCGGCCACGGACC | | | | - | 26 | |  |
| 113415 | 113427 | | 13 | GGACCGGTGGTGG | | | | + | 30 | |  |
| 113483 | 113495 | | 13 | CCACCGCCTCACC | | | | - | 30 | |  |
| 114250 | 114267 | | 18 | CCGTAGGCCTGCGCCTCC | | | | - | 23 | |  |
| 114389 | 114408 | | 20 | CCTTGGCGCCAGTGGCCACC | | | | - | 20 | |  |
| 114611 | 114627 | | 17 | CCTCCTGATGTGCCTCC | | | | - | 24 | |  |
| 114763 | 114782 | | 20 | CCACCGGAGGAGCCCGCTCC | | | | - | 20 | |  |
| 115402 | 115414 | | 13 | CCTACCTCCAGCC | | | | - | 30 | |  |
| 115744 | 115763 | | 20 | CCACGCTTTCCCGTGCCTCC | | | | - | 20 | |  |
| 115767 | 115780 | | 14 | GGTTGCGGTGGTGG | | | | + | 29 | |  |
| 116522 | 116539 | | 18 | CCTCCATAACGCCTTGCC | | | | - | 23 | |  |
| 117727 | 117745 | | 19 | CCGCACCGCCCCTTCTTCC | | | | - | 21 | |  |
| 118683 | 118696 | | 14 | CCTACCACCAGGCC | | | | - | 29 | |  |
| 118777 | 118787 | | 11 | CCTCCTCCCCC | | | | - | 33 | |  |
| 119641 | 119657 | | 17 | GGAGGATAGGCAGCAGG | | | | + | 24 | |  |
| 119882 | 119900 | | 19 | GGAATAGGAAAAGGGCAGG | | | | + | 21 | |  |
| 120218 | 120232 | | 15 | GGGGGAATGGTGTGG | | | | + | 27 | |  |
| 123809 | 123822 | | 14 | GGATAGGTGGGCGG | | | | + | 29 | |  |
| 124653 | 124669 | | 17 | GGTATTGAGGACGGTGG | | | | + | 24 | |  |
| 124778 | 124791 | | 14 | CCTTATCCCCCTCC | | | | - | 29 | |  |
| 124928 | 124944 | | 17 | CCACCCTCCCAAATACC | | | | - | 24 | |  |
| 125054 | 125071 | | 18 | GGTGGCCTTCCAGGTTGG | | | | + | 23 | |  |
| 125322 | 125335 | | 14 | CCCCCGGCCTTGCC | | | | - | 29 | |  |
| 125882 | 125898 | | 17 | GGCGCTGGAGGGGCTGG | | | | + | 24 | |  |
| 125997 | 126011 | | 15 | GGGGGCCTGGTGAGG | | | | + | 27 | |  |
| 127208 | 127224 | | 17 | GGACTGCAGGGAGGTGG | | | | + | 24 | |  |
| 127252 | 127267 | | 16 | GGAAATGGTGGTGAGG | | | | + | 26 | |  |
| 127293 | 127306 | | 14 | GGAGGAGGTTTAGG | | | | + | 29 | |  |
| 128004 | 128017 | | 14 | GGAGGAGGCGCAGG | | | | + | 29 | |  |
| 128082 | 128095 | | 14 | GGTGGTCTGGCTGG | | | | + | 29 | |  |
| 128424 | 128442 | | 19 | CCATCCAAAGTAGCCAACC | | | | - | 21 | |  |
| 128631 | 128646 | | 16 | GGACTCTGGAGGGTGG | | | | + | 26 | |  |
| 128734 | 128751 | | 18 | GGCGGTGAAAGGGCAAGG | | | | + | 23 | |  |
| 129781 | 129794 | | 14 | CCACGCCTCCGACC | | | | - | 29 | |  |
| 130024 | 130042 | | 19 | CCACCCCGCCGTGAAGGCC | | | | - | 21 | |  |
| 130252 | 130271 | | 20 | GGTAAAGGTATATAAGGGGG | | | | + | 20 | |  |
| 131142 | 131161 | | 20 | GGGGGAACCAACGGCGTCGG | | | | + | 20 | |  |
| 131194 | 131210 | | 17 | CCATTCTTACCACCGCC | | | | - | 24 | |  |
| 131265 | 131281 | | 17 | CCTCCTACCATTACACC | | | | - | 24 | |  |
| 131809 | 131826 | | 18 | GGACCGGCCAGCGGAAGG | | | | + | 23 | |  |
| 131994 | 132012 | | 19 | GGAGGGAAAAGGGCGCAGG | | | | + | 21 | |  |
| 132085 | 132104 | | 20 | CCAACCCAAGCGGGCCCTCC | | | | - | 20 | |  |
| 133036 | 133051 | | 16 | CCTCCCACCTCTATCC | | | | - | 26 | |  |
| 133092 | 133108 | | 17 | GGAGGAAAGGATGCAGG | | | | + | 24 | |  |
| 133202 | 133218 | | 17 | GGACGGCCAGCGGGCGG | | | | + | 24 | |  |
| 134756 | 134771 | | 16 | GGCGGTGGTGACGCGG | | | | + | 26 | |  |
| 135665 | 135677 | | 13 | CCTCCGGGCCGCC | | | | - | 30 | |  |
| 136701 | 136714 | | 14 | GGTGGAAGGCCAGG | | | | + | 29 | |  |
| 136871 | 136885 | | 15 | CCATAACCCCCGGCC | | | | - | 27 | |  |
| 138195 | 138208 | | 14 | GGGGGCCGGGATGG | | | | + | 29 | |  |
| 138318 | 138328 | | 11 | GGAGGGGGTGG | | | | + | 33 | |  |
| 139051 | 139069 | | 19 | CCTATCCAAAACCACAACC | | | | - | 21 | |  |
| 140318 | 140333 | | 16 | GGTTGGCAGGGATTGG | | | | + | 26 | |  |
| 141267 | 141281 | | 15 | GGCAGAGGGCGGCGG | | | | + | 27 | |  |
| 142044 | 142058 | | 15 | CCCCCTGGCCTATCC | | | | - | 27 | |  |
| 142203 | 142217 | | 15 | GGACGGGGAAGGAGG | | | | + | 27 | |  |
| 142908 | 142924 | | 17 | GGAAAAGGTTGTGGTGG | | | | + | 24 | |  |
| 142973 | 142992 | | 20 | CCATACCATCGGGCCGGTCC | | | | - | 20 | |  |
| 142988 | 143005 | | 18 | GGTCCAGGGGTGTGGCGG | | | | + | 23 | |  |
| 144283 | 144299 | | 17 | GGCTTCTTGGGGGTCGG | | | | + | 24 | |  |
| 144356 | 144371 | | 16 | GGGGGCTGAGAGGCGG | | | | + | 26 | |  |
| 145208 | 145226 | | 19 | CCATTCCTCCTCTTGGGCC | | | | - | 21 | |  |
| 145559 | 145578 | | 20 | CCGCTGGCCCGCCAGTTGCC | | | | - | 20 | |  |
| 147141 | 147156 | | 16 | GGAGCCATGGAGGCGG | | | | + | 26 | |  |
| 147618 | 147634 | | 17 | CCCCCACCGAGATTACC | | | | - | 24 | |  |
| 148135 | 148152 | | 18 | CCTGACCTCGACCATTCC | | | | - | 23 | |  |
| 149688 | 149702 | | 15 | GGAAGGACCTGGGGG | | | | + | 27 | |  |
| 150061 | 150091 | | 31 | GGGTAGATTTGGGAGCACGGGCGAGGCCGGG | | | | + | 51 | |  |
| 150524 | 150541 | | 18 | GGTGGTGAAGGGGAATGG | | | | + | 23 | |  |
| 151053 | 151069 | | 17 | CCATTCCCAGCCCTACC | | | | - | 24 | |  |
| 151931 | 151948 | | 18 | CCGTGATCCCACCAAGCC | | | | - | 23 | |  |
| 153181 | 153203 | | 23 | CCCATTCACCCTCGGGCCCTCCC | | | | - | 61 | |  |
| 153293 | 153309 | | 17 | GGTGTCGGTTGGCAAGG | | | | + | 24 | |  |
| 156230 | 156247 | | 18 | GGTAGGTTGGGGTGTTGG | | | | + | 23 | |  |
| 157070 | 157088 | | 19 | GGTATTGGTGGAACATTGG | | | | + | 21 | |  |
| 157431 | 157448 | | 18 | GGTATTCTGGAGGAAAGG | | | | + | 23 | |  |
| 159035 | 159045 | | 11 | CCTCCTCCTCC | | | | - | 33 | |  |
| 159829 | 159847 | | 19 | GGTCTTTGGGCTGTGGTGG | | | | + | 21 | |  |
| 160086 | 160103 | | 18 | CCAAACCACCAAAGGGCC | | | | - | 23 | |  |
| 163970 | 163983 | | 14 | CCCCCTCCTTAACC | | | | - | 29 | |  |
| 164606 | 164619 | | 14 | CCGCCACCTGCTCC | | | | - | 29 | |  |
| 164712 | 164722 | | 11 | CCGCCGCCCCC | | | | - | 33 | |  |
| 165833 | 165851 | | 19 | GGTTTATGGTGGCATGAGG | | | | + | 21 | |  |
| 167784 | 167796 | | 13 | CCGCCGCCCAGCC | | | | - | 30 | |  |

## Supplementary Table S4. Oligonucleotides and primer sequences.

| Primer | | | |  | | Sequence (5′ to 3′) * |
| --- | --- | --- | --- | --- | --- | --- |
| **Q-PCR**  E165R-F  E165R-R  ASFV-F  ASFV-R  ASFV-probe | | | |  | | CTGTTGCTCCGACCTAGTGCTTF  TACTGCTTCTCGCCATGATTTT  CTGCTCATGGTATCAATCTTATCGA  GATACCACAAGATCRGCCGT  FAM-CCACGGGAGGAATACCAACCCAGTG-TAMRA |
| **CD and fluorescent assay** | | | | | | |
| G4-WT | | |  | | **GGG**TAGATTT**GGG**AGCAC**GGG**CGAGGCC**GGG** | |
| G4-Mut | | |  | | GAGTAGATTTGAGAGCACGAACGAGGCCAAG | |
| RNA-G4 | | |  | | **GGG**UAGAUUU**GGG**AGCAC**GGG**CGAGGCC**GGG** | |
| RNA-G4-Mut | | |  | | GAGUAGAUUUGAGAGCACGAACGAGGCCAAG | |
| c-myc | | |  | | TGGGGAGGGTGGGGAGGGTGGGGAAGG | |
| Tel22 | | |  | | AGGGTTAGGGTTAGGGTTAGGG | |
| Tel22R | | |  | | AGGGUUAGGGUUAGGGUUAGGG | |
| **PAGE**  G4-WT | | |  | | FAM-**GGG**TAGATTT**GGG**AGCAC**GGG**CGAGGCC**GGG** | |
| G4-Mut | | |  | | FAM-GAGTAGATTTGAGAGCACGAACGAGGCCAAG | |
| Tel22-WT | | |  | | FAM-AGGGTTAGGGTTAGGGTTAGGG | |
| Tel22-Mut | | |  | | FAM-AGGGTTAGGGTTAGGGTTAGGG | |
| Marker-25 nt | | |  | | FAM-TTTTTTTTTTTTTTTTTTTTTTTTT | |
| Marker-35 nt | | |  | | FAM-TTTTTTTTTTTTTTTTTTTTTTTTTTTTTTTTTTT | |
| **FRET melting**  DNA-G4 |  | | FAM-**GGG**TAGATTT**GGG**AGCAC**GGG**CGAGGCC**GGG**-TAMAR | | | |
| F21T |  | | FAM-**GGG**TTA**GGG**TTA**GGG**TTA**GGG**-TAMAR | | | |
| **Taq polymerase termination assay** | | | | | | |
| G4-WT-Template | | TTTTT**GGG**TAGATTT**GGG**AGCAC**GGG**CGAGGCC**GGG**TTTTTCGCA*CTGAGCGAAGATACGGAGCCACGCCA* | | | | |
| G4-Mut-Template | | TTTTTGAGTAGATTTGAGAGCACGAACGAGGCCAAGTTTTTCGCA*CTGAGCGAAGATACGGAGCCACGCCA* | | | | |
| Primer | | FAM-TGGCGTGGCTCCGTATCTTCGCTCAG | | | | |

* Gs that participate in G4 folding are shown in bold, and the mutated bases are underlined. For the Taq polymerase termination experiment, the complementary primer region is indicated by italics.
