# Supplementary material for: Identification of a conserved G-quadruplex within the E165R of African swine fever virus (ASFV) as a potential antiviral target
Source: J Biol Chem. 2024 Jun 7;300(7):107453. doi: 10.1016/j.jbc.2024.107453 (PMC11261444; doi:10.1016/j.jbc.2024.107453)
Supplement: Supporting Figures [file mmc2.doc]

**Supplementary Figure legends**

**Figure S1.** Formation of the E165R G4 reduces the efficiency of the in vitro EGFP expression. The plasmids pEGFP-G4 and pEGFP-G4-Mut were transfected into HEK293T cells, respectively. After 48 hours of transfection, the EGFP(left) and DAPI(middle) were detected using a confocal microscope.The pEGFP-N1 vector was used as control. The scale bar was 20 μm.

**Figure S2.** Effects of the etoposide on the proliferation of ASFV in Vero cells. Vero cells were pretreated with various concentrations of etoposide (0 μM, 5 μM, 10 μM, 20 μM and 30 μM) and then infected with virus (ASFV-GFP) at an MOI of 1, followed by the replacement of the medium containing the corresponding concentration of etoposide. After 36 hours of infection, fluorescence was observed under a fluorescence microscope. The scale bar was 200 μm.
